# Immune checkpoint inhibitor-associated autoimmune encephalitis and other neurological immune-mediated adverse events: a pharmacovigilance study using the FAERS and JADER

**DOI:** 10.3389/fonc.2025.1621045

**Published:** 2025-07-17

**Authors:** Xiaomeng Di, Xiaohong Shi, Feng Gai, Jiawei Wang

**Affiliations:** Department of Neurology, Beijing Tongren Hospital, Capital Medical University, Beijing, China

**Keywords:** immune checkpoint inhibitor, autoimmune encephalitis, immune-related adverse events, pharmacovigilance, FAERS

## Abstract

**Background:**

Immune checkpoint inhibitor (ICI)-associated neurological immune-related adverse events (NAEs) are rare but serious side effects, of which autoimmune encephalitis (AIE) is a potentially fatal central nervous system disorder requiring more attention.

**Methods:**

We performed a retrospective disproportionality analysis of NAE reports in the FDA Adverse Event Reporting System (FAERS) and the Japanese Adverse Event Reporting Database (JADER) from 2004 to 2024, utilizing reporting odds ratio (ROR), proportional reporting ratio (PRR), the Bayesian confidence propagation neural network BCPNN, and the multi-item gamma Poisson shrinker (MGPS) for signal detection.

**Results:**

In total, 3,999 reports of ICI-associated NAEs were identified from the FAERS database, of which 1,998 reports were AIE. 1,558,251 reports of AEs were collected from the JADER database, which contained 890 AIE reports. ICIs, including pembrolizumab, nivolumab, atezolizumab, ipilimumab, and durvalumab, were identified among the top 30 agents in both databases, demonstrating significant signals across all 4 algorithms. Except for noninfectious myelitis, acute disseminated encephalomyelitis, and multiple sclerosis, positive signals were detected in all other preferred terms (PTs). These NAEs accounted for 23.7% of total mortality, with myasthenia gravis (MG) exhibiting the highest mortality rate at 30.63%. Specific PTs, such as aseptic meningitis, AIE, chronic inflammatory demyelinating polyradiculoneuropathy, Guillain-Barré syndrome, MG, myelitis, and immune-related myopathy, were associated with the severity of outcomes, showing significant statistical differences between severe and non-severe cases (p < 0.05).

**Conclusion:**

Our study found a notable correlation between ICIs and AIE and other specific NAEs, highlighting the demographic characteristics, time to onset, and disease severity of ICI-induced NAEs, thereby facilitating the timely recognition and treatment of these ICI therapy-related complications.

## 1 Introduction

Immune checkpoints constitute a critical group of regulatory molecules expressed on immune cells, pivotal in modulating immune response activation. Notable examples include cytotoxic T-lymphocyte antigen 4 (CTLA-4), programmed death protein 1 (PD-1) and its ligand, programmed death ligand 1 (PD-L1), as well as lymphocyte activation gene-3 (LAG-3). Commonly used immune checkpoint inhibitors (ICIs) encompass the CTLA-4 inhibitor, including ipilimumab, PD-1 inhibitors such as pembrolizumab, nivolumab, and cemiplimab, as well as PD-L1 inhibitors, including atezolizumab, durvalumab, and avelumab. More than 10 ICI drugs have been approved for marketing by the Food and Drug Administration (FDA). Moreover, these agents have been widely utilized in clinical settings to treat multiple malignancies, such as melanoma, non-small cell lung cancer, head and neck squamous cell carcinoma, Hodgkin’s lymphoma, bladder cancer, and colorectal cancer. In clinical practice, CTLA-4 inhibitors are frequently combined with PD-1/PD-L1 inhibitors. ICI therapy significantly advances tumor immunotherapy, augmenting the body’s defenses against cancer (1, 2).

Although ICIs have high efficacy compared with traditional chemotherapies and can effectively improve the survival rate of cancer patients, their adverse effects should not be neglected. These pharmacological agents are associated with various immune-related adverse events (irAEs). These events arise from inappropriate stimulation of the immune system, inadvertently targeting normal tissues, predominantly affecting the skin, gastrointestinal tract, lungs, thyroid, and musculoskeletal system (3). Neurological irAEs (NAEs) are relatively uncommon, with incidence rates of 3.8% associated with CTLA-4 inhibitors, 6% with PD-1 inhibitors, and 12% with combination therapies. ICI-induced NAEs include central nervous system (CNS) and peripheral nervous system (PNS) irAEs. Documented NAEs encompass autoimmune encephalitis (AIE), aseptic meningitis (4), Guillain-Barré syndrome (GBS), chronic inflammatory demyelinating polyradiculoneuropathy (CIDP), myasthenia gravis (MG), and CNS demyelinating diseases such as neuromyelitis optica spectrum disorder (NMOSD) (5, 6), myelin oligodendrocyte glycoprotein antibody-associated disease (MOGAD) (7), and CNS vasculitis (8).

AIE is a CNS disorder characterized by antibodies targeting neuronal antigens. These antibodies predominantly target neuronal surface antigens, intracellular antigens, or synaptic proteins, resulting in alterations and damage to neural function. The pathogenesis of AIE is complex, involving various factors such as viral infections, vaccinations, and neoplasms (9). Tumor-associated AIE, particularly in the context of paraneoplastic neurological syndromes, has been extensively studied due to the resemblance between tumor cell antigens and neuronal antigens. As such, this triggers immune system attacks on the nervous system. Recent research indicates that the ICI therapy can also trigger AIE, although this occurrence is uncommon (10). In such cases, patients may require the discontinuation of ICI therapy and the initiation of immunosuppressive treatment to manage symptoms. Thus, the early identification and diagnosis of ICI-related AIE, along with balancing tumor and AIE treatment, present significant challenges.

There is still a lack of comprehensive studies on NAEs associated with ICIs. Recently, comprehensive real-world drug adverse event databases, such as the United States FDA Adverse Event Reporting System (FAERS) and the Japanese Adverse Drug Event Report (JADER) of the Pharmaceuticals and Medical Devices Agency, comprising spontaneous adverse event reports, are essential resources and analytical instruments in pharmacovigilance. Thorough examination of these databases enables researchers to gain a comprehensive insight into adverse drug event profiles, thereby furnishing crucial safety insights for clinical application. These discoveries improve drug safety protocols and provide empirical support for risk mitigation strategies and policy formulation in pharmacotherapy. Thus, we conducted a disproportionality analysis focusing on AIE and other NAEs associated with ICIs, utilizing real-world data from FAERS and JADER from 2004 to 2024.

## 2 Materials and methods

### 2.1 Data resources

Data were obtained from the FAERS website (https://fis.fda.gov/extensions/FPD-QDE-FAERS/FPD-QDE-FAERS.html), spanning 20 years from the first quarter of 2004 (2024Q1) to the fourth quarter of 2024 (2024Q4), and JADER website (https://www.pmda.go.jp/safety/info-services/drugs/adr-info/suspected-adr/0003.html) from the first quarter of 2004 (2024Q1) to the third quarter of 2024 (2024Q3). The data tables in the FAERS database include DEMO (demographic and administrative information), DRUG (drug information), INDI (indications for drug administration), REAC (coded AEs), and OUTC (outcomes of patients). In the JADER database, we utilized the following 3 data tables for analysis: DEMO, DRUG, and REAC.

### 2.2 Data extraction

Duplicate reports were eliminated by utilizing a unique safety report identifier, “primaryID”, assigned to each case. AEs in the REAC table were classified using the “Preferred Term” (PT) from the Medical Dictionary for Regulatory Activities (MedDRA). In this research, we examine the following PTs in MedDRA 26.0: AIE (including autoimmune encephalopathy, encephalitis autoimmune, immune-mediated encephalitis, noninfective encephalitis), other NAEs include PNS immune-mediated diseases (including Guillain-Barre syndrome, Miller Fisher syndrome, acute motor axonal neuropathy, acute motor-sensory axonal neuropathy, chronic inflammatory demyelinating polyradiculoneuropathy), neuromuscular junction dysfunction (including immune-mediated myasthenia gravis, myasthenia gravis, myasthenia gravis crisis, myasthenic syndrome, ocular myasthenia), immune-related myopathy (including autoimmune myositis, dermatomyositis, immune-mediated myositis, myositis, necrotizing myositis, polymyositis), myelitis (including immune-mediated myelitis, myelitis, myelitis transverse, noninfectious myelitis), CNS demyelinating diseases (including neuromyelitis optica spectrum disorder, acute disseminated encephalomyelitis, multiple sclerosis, primary progressive multiple sclerosis, progressive multiple sclerosis, progressive relapsing multiple sclerosis, relapsing multiple sclerosis, relapsing-remitting multiple sclerosis, secondary progressive multiple sclerosis, myelin oligodendrocyte glycoprotein antibody-associated disease), aseptic meningitis (including meningitis aseptic, meningitis noninfective), and CNS vasculitis (central nervous system vasculitis).

**Figure 1:**
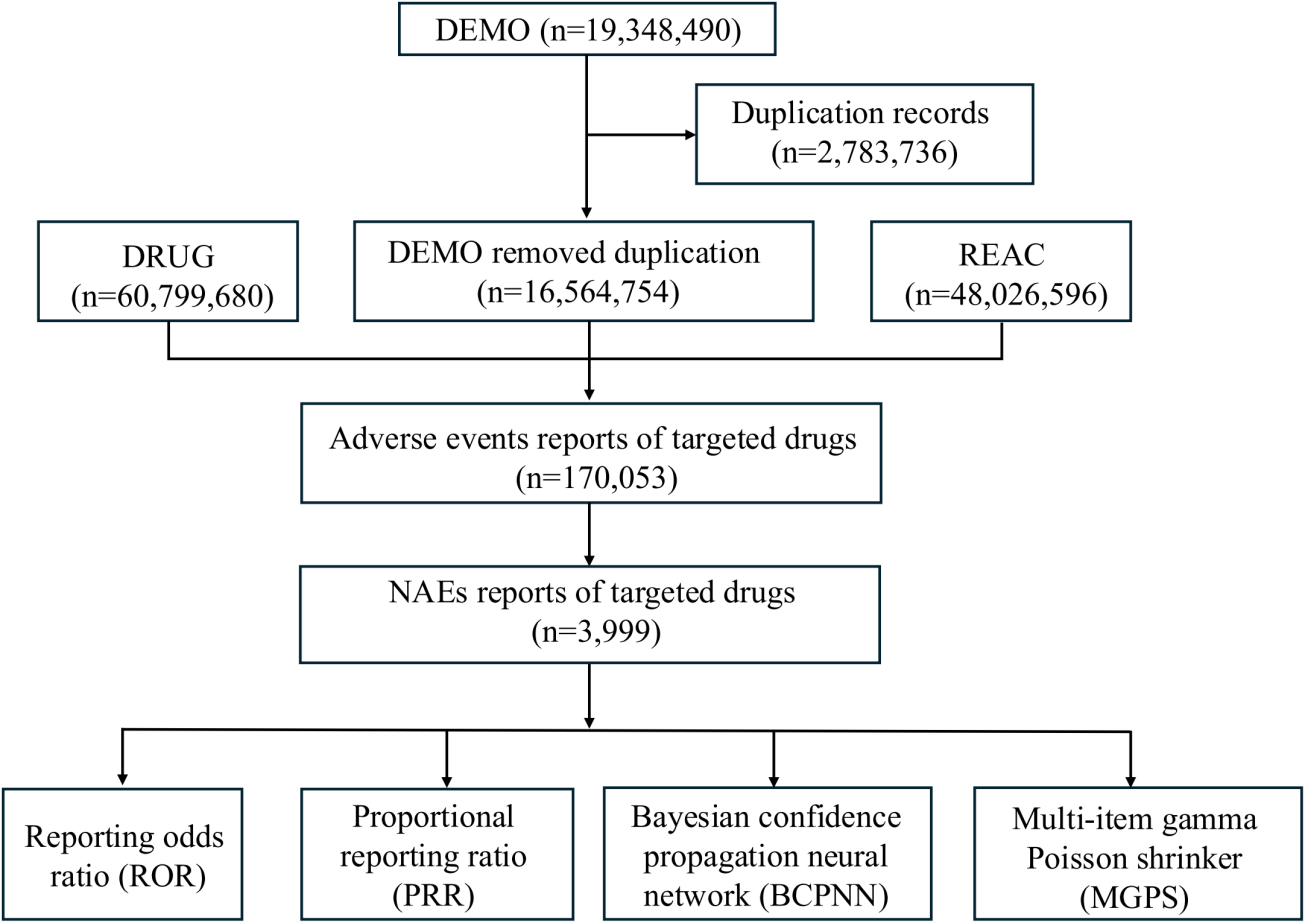
Flowchart of data extraction and disproportionality analysis of ICI-related NAEs in the FAERS database from 2004 Q1 to 2024 Q4.

In this study, we selected the following ICIs for research: ipilimumab (IPI), pembrolizumab (PEM), nivolumab (NIV), cemiplimab (CEM), atezolizumab (ATE), durvalumab (DUR), and avelumab (AVE). Due to inconsistent registration of drug names in the FAERS database, we employed both generic and brand names of ICIs as search keywords. The DRUG table categorizes drug role codes into 4 groups based on their involvement in AEs: primary suspect (PS), secondary suspect (SS), concomitant (C), and interaction (I). Notably, this research targeted the PS category as the study population. **Figure 1** shows the flowchart for screening ICI-associated NAEs.

According to the OUTC table, death (DE) outcomes were categorized as serious. In contrast, other outcomes, including life-threatening (LT), hospitalization (HO), disability (DS), congenital anomaly (CA), required intervention (RI), and other outcomes (OT), were classified as non-serious.

### 2.3 Statistical analysis

In this study, we employ 4 algorithms for the detection of adverse event signals: the reporting odds ratio (ROR) (11), the proportional reporting ratio (PRR) (12), the Bayesian confidence propagation neural network (BCPNN) (13), and the multi-item gamma Poisson shrinker (MGPS) (14). The formulas and signal detection criteria for these 4 algorithms are listed in **Supplementary Table S1**. Notably, these are based on a four-cell table of disproportional methods. A signal of NAEs is considered present when at least one of these algorithms meets the established criteria, indicating a statistical association between drug treatment and AEs. All statistical analyses were conducted using R software (version 4.2.2). Correlations were evaluated through the non-parametric Spearman correlation test. Furthermore, we performed a Chi-square test to compare each PT’s serious and non-serious outcome groups. Statistical significance was considered when the p-value was < 0.05.

## 3 Results

### 3.1 The top 30 drugs with the highest number of reported AIE cases in the FAERS and JADER databases, respectively

We utilize the FAERS and JADER databases to identify the top 30 drugs associated with AIE. A disproportionality analysis was performed to explore the potential association between drugs and AIE. Notably, the number of reported cases may exceed the count of target AEs, as a single case can encompass multiple such events.

The FAERS database contains 16,564,754 reports of AEs after removing duplication from 2004Q1 to 2024Q4, of which 1,998 reports were AIE. The JADER database contains 1,558,251 reports of AEs from 2004Q1 to 2024Q3, of which 890 reports were AIEs. Signal values determined by all 4 algorithms are listed in the **Table 1**.

**Table 1:** Signal strength of AIE reports for Top 30 drugs in the FAERS and JADER databases.

Database	Drug	N	ROR (95%CI)	PRR (χ2)	EBGM (EBGM05)	IC (IC025)
FAERS	Pembrolizumab	166	33.79 (28.83 - 39.61)	33.76 (4841.34)	31.05 (27.19)	4.96 (4.72)
	Nivolumab	158	26.53 (22.55 - 31.21)	26.51 (3573.71)	24.5 (21.39)	4.61 (4.38)
	Adalimumab	112	1.68 (1.39 - 2.04)	1.68 (29.28)	1.64 (1.4)	0.72 (0.44)
	Atezolizumab	87	44.57 (35.95 - 55.26)	44.5 (3539.63)	42.62 (35.6)	5.41 (5.1)
	Levetiracetam	81	15.45 (12.37 - 19.3)	15.45 (1050.37)	14.86 (12.34)	3.89 (3.57)
	Rituximab	73	4.42 (3.5 - 5.58)	4.42 (185.99)	4.29 (3.53)	2.1 (1.76)
	Mycophenolate mofetil	51	7.95 (6.02 - 10.5)	7.95 (301.83)	7.77 (6.16)	2.96 (2.55)
	Ipilimumab	47	29.22 (21.88 - 39.03)	29.19 (1249.64)	28.53 (22.39)	4.83 (4.41)
	Dimethyl fumarate	45	4.23 (3.15 - 5.69)	4.23 (108.66)	4.16 (3.25)	2.06 (1.63)
	Prednisolone	40	6.23 (4.56 - 8.53)	6.23 (172.23)	6.13 (4.72)	2.62 (2.16)
	IVIG	36	4.09 (2.94 - 5.68)	4.08 (82.36)	4.03 (3.06)	2.01 (1.53)
	Natalizumab	35	2.43 (1.74 - 3.39)	2.43 (28.9)	2.4 (1.82)	1.27 (0.78)
	Tacrolimus	35	5.16 (3.69 - 7.2)	5.16 (115.24)	5.08 (3.84)	2.35 (1.86)
	Lenvatinib	33	13.22 (9.37 - 18.65)	13.21 (366.47)	13.01 (9.76)	3.7 (3.2)
	Fingolimod	32	3.28 (2.31 - 4.65)	3.28 (49.96)	3.25 (2.42)	1.7 (1.19)
	Infliximab	30	1.1 (0.76 - 1.57)	1.1 (0.25)	1.09 (0.81)	0.13 (-0.39)
	Carboplatin	26	5.12 (3.48 - 7.54)	5.12 (85.08)	5.07 (3.67)	2.34 (1.78)
	Olanzapine	26	3.93 (2.67 - 5.78)	3.93 (56.03)	3.89 (2.81)	1.96 (1.4)
	Methotrexate	24	1.26 (0.84 - 1.88)	1.26 (1.25)	1.25 (0.9)	0.33 (-0.25)
	Durvalumab	23	25.04 (16.6 - 37.78)	25.02 (524.34)	24.75 (17.54)	4.63 (4.04)
	Interferon beta-1a	22	1.29 (0.85 - 1.96)	1.29 (1.4)	1.28 (0.9)	0.36 (-0.24)
	Etanercept	16	0.32 (0.19 - 0.52)	0.32 (23.1)	0.32 (0.21)	-1.62 (-2.33)
	Bevacizumab	16	2.15 (1.31 - 3.51)	2.15 (9.73)	2.14 (1.42)	1.1 (0.39)
	Risperidone	15	2.17 (1.31 - 3.61)	2.17 (9.42)	2.16 (1.41)	1.11 (0.39)
	Lamotrigine	14	2.13 (1.26 - 3.61)	2.13 (8.39)	2.13 (1.37)	1.09 (0.34)
	Ocrelizumab	13	2.11 (1.22 - 3.64)	2.11 (7.56)	2.1 (1.33)	1.07 (0.3)
	Tocilizumab	13	1.37 (0.8 - 2.37)	1.37 (1.31)	1.37 (0.87)	0.46 (-0.32)
	Glatiramer acetate	12	2.42 (1.37 - 4.26)	2.42 (9.91)	2.41 (1.5)	1.27 (0.47)
	Alemtuzumab	11	4.19 (2.31 - 7.57)	4.18 (26.52)	4.17 (2.54)	2.06 (1.22)
	Daclizumab	10	42.39 (22.76 - 78.95)	42.33 (401.54)	42.12 (25.04)	5.4 (4.52)
JADER	Nivolumab	147	12.67 (10.45 - 15.35)	12.63 (1115.79)	9.24 (7.62)	3.21 (1.54)
	Pembrolizumab	113	12.73 (10.33 - 15.7)	12.7 (944.92)	10.07 (8.17)	3.33 (1.66)
	Ipilimumab	94	11.65 (9.31 - 14.58)	11.62 (742.36)	9.64 (7.7)	3.27 (1.6)
	Atezolizumab	50	13.84 (10.33 - 18.55)	13.79 (534.42)	12.52 (9.34)	3.65 (1.98)
	Recombinant adsorbed bivalent HPV-like particle vaccine	30	11.14 (7.7 - 16.12)	11.1 (259.5)	10.5 (7.26)	3.39 (1.72)
	Coronavirus (SARS-CoV-2) RNA vaccine	29	0.8 (0.55 - 1.17)	0.8 (1.34)	0.81 (0.56)	-0.3 (-1.97)
	Lenvatinib	27	7.15 (4.85 - 10.53)	7.13 (134.76)	6.8 (4.61)	2.77 (1.1)
	Durvalumab	23	9.33 (6.13 - 14.18)	9.3 (162.67)	8.92 (5.87)	3.16 (1.49)
	Carboplatin	18	2.38 (1.48 - 3.8)	2.38 (13.82)	2.33 (1.45)	1.22 (-0.45)
	Tacrolimus	16	1.82 (1.11 - 3)	1.82 (5.74)	1.8 (1.09)	0.84 (-0.83)
	Bevacizumab	13	1.35 (0.78 - 2.34)	1.35 (1.16)	1.34 (0.77)	0.42 (-1.25)
	Mycophenolate mofetil	12	2.92 (1.65 - 5.19)	2.92 (14.82)	2.88 (1.62)	1.52 (-0.15)
	Etoposide	12	2.95 (1.67 - 5.24)	2.95 (15.12)	2.91 (1.64)	1.54 (-0.13)
	Prednisolone	11	0.57 (0.32 - 1.04)	0.57 (3.4)	0.58 (0.32)	-0.78 (-2.45)
	Cyclosporine	11	1.96 (1.08 - 3.57)	1.96 (5.07)	1.94 (1.07)	0.96 (-0.72)
	Influenza HA vaccine	11	5.52 (3.04 - 10.05)	5.52 (39.8)	5.42 (2.98)	2.44 (0.77)
	Recombinant precipitated tetravalent HPV-like particle vaccine	11	10.69 (5.87 - 19.45)	10.66 (94.18)	10.45 (5.74)	3.38 (1.71)
	Cisplatin	11	1.52 (0.83 - 2.76)	1.52 (1.89)	1.5 (0.83)	0.59 (-1.08)
	Paclitaxel	10	1.31 (0.7 - 2.44)	1.31 (0.7)	1.3 (0.69)	0.38 (-1.29)
	Methotrexate	9	0.61 (0.32 - 1.18)	0.61 (2.18)	0.62 (0.32)	-0.69 (-2.36)
	Cyclophosphamide	7	1.05 (0.5 - 2.21)	1.05 (0.02)	1.05 (0.5)	0.07 (-1.6)
	Freeze-dried live attenuated varicella vaccine	7	26.04 (12.31 - 55.08)	25.84 (164.86)	25.49 (12.05)	4.67 (3)
	Freeze-dried live attenuated mumps vaccine	6	13.98 (6.24 - 31.33)	13.93 (71.15)	13.77 (6.15)	3.78 (2.11)
	Adsorbed 13-valent pneumococcal conjugate vaccine	5	2.94 (1.22 - 7.09)	2.94 (6.32)	2.92 (1.21)	1.54 (-0.13)
	Dried Haemophilus influenzae type b vaccine	5	2.87 (1.19 - 6.93)	2.87 (6.03)	2.85 (1.18)	1.51 (-0.16)
	Tremelimumab	5	7.15 (2.96 - 17.28)	7.14 (26.14)	7.08 (2.93)	2.82 (1.15)
	Cabozantinib	5	2.8 (1.16 - 6.76)	2.8 (5.73)	2.78 (1.15)	1.48 (-0.19)
	Levetiracetam	4	2.19 (0.82 - 5.86)	2.19 (2.56)	2.18 (0.81)	1.12 (-0.55)
	Recombinant precipitated 9-valent HPV-like particle vaccine (yeast-derived)	4	22.05 (8.21 - 59.18)	21.9 (79.18)	21.73 (8.1)	4.44 (2.77)
	Carbamazepine	3	0.79 (0.25 - 2.45)	0.79 (0.17)	0.79 (0.25)	-0.34 (-2.02)

AIE, autoimmune encephalitis; N, Number of cases of adverse events; HPV, Human papillomavirus.

In both databases, ICIs strongly correlated with AIE. In the TOP 30 drugs of FAERS database, the following ICI drugs showed significant signals: pembrolizumab (N= 166, ROR= 33.79, 95%CI [28.83~39.61], PRR= 33.76, χ2 = 4841.34, EBGM= 31.05, EBGM05 = 27.19, IC= 4.96, IC025 = 4.72), nivolumab (N= 158, ROR= 26.53, 95%CI [22.55~31.21], PRR= 26.51, χ2 = 3573.71, EBGM= 24.5, EBGM05 = 21.39, IC= 4.61, IC025 = 4.38), atezolizumab (N= 87, ROR= 44.57, 95%CI [35.95~55.26], PRR= 44.5, χ2 = 3539.63, EBGM= 42.62, EBGM05 = 35.6, IC= 5.41, IC025 = 5.1), ipilimumab (N= 47, ROR= 29.22, 95% CI [21.88~39.03], PRR= 29.19, χ2 = 1249.64, EBGM= 28.53, EBGM05 = 22.39, IC= 4.83, IC025 = 4.41), and durvalumab (N= 23, ROR= 25.04, 95%CI [16.6~37.78], PRR= 25.02, χ2 = 524.34, EBGM= 24.75, EBGM05 = 17.54, IC= 4.63, IC025 = 4.04).

In the TOP 30 drugs of JADER database, the following ICI drugs showed significant signals: nivolumab (N= 147, ROR= 12.67, 95%CI [10.45~15.35], PRR= 12.63, χ2 = 1115.79, EBGM= 9.24, EBGM05 = 7.62, IC= 3.21, IC025 = 1.54), pembrolizumab (N= 113, ROR= 12.73, 95%CI [10.33~15.7], PRR= 12.7, χ2 = 944.92, EBGM= 10.07, EBGM05 = 8.17, IC= 3.33, IC025 = 1.66), ipilimumab (N= 94, ROR= 11.65, 95%CI [9.31~14.58], PRR= 11.62, χ2 = 742.36, EBGM= 9.64, EBGM05 = 7.7, IC= 3.27, IC025 = 1.6), atezolizumab (N= 50, ROR= 13.84, 95%CI [10.33~18.55], PRR= 13.79, χ2 = 534.42, EBGM= 12.52, EBGM05 = 9.34, IC= 3.65, IC025 = 1.98), durvalumab (N= 23, ROR= 9.33, 95%CI [6.13~14.18], PRR= 9.3, χ2 = 162.67, EBGM= 8.92, EBGM05 = 5.87, IC= 3.16, IC025 = 1.49), and Tremelimumab (N= 5, ROR= 7.15, 95%CI [2.96~17.28], PRR= 7.14, χ2 = 26.14, EBGM= 7.08, EBGM05 = 2.93, IC= 2.82, IC025 = 1.15).

### 3.2 Demographic characteristics of AIE among ICI-treated patients in the FAERS and JADER

**Table 2** summarizes the clinical characteristics and reporting patterns of autoimmune encephalitis (AIE) associated with 5 ICI drugs in 2 pharmacovigilance databases, FAERS and JADER. The analysis includes the number of reported cases, gender distribution, age distribution, reporter types, outcomes (death vs. non-death), and reporting year distribution for each of the ICI drugs: ipilimumab, pembrolizumab, nivolumab, atezolizumab, and durvalumab. Cempilimab and avelumab were excluded due to insufficient data.

**Table 2:** Characteristics of AIE correlated with ICIs in the FAERS and JADER databases.

Characteristics, number	FAERS					JADER				
	IPI	PEM	NIV	ATE	DUR	IPI	PEM	NIV	ATE	DUR
N	N=47	N=164	N=158	N=86	N=23	N=93	N=112	N=142	N=50	N=23
Gender										
Female	20 (42.6%)	79 (48.2%)	56 (35.4%)	36 (41.9%)	10 (43.5%)	30 (32.3%)	52 (46.4%)	39 (27.5%)	10 (20.0%)	3 (13.0%)
Male	25 (53.2%)	79 (48.2%)	91 (57.6%)	45 (52.3%)	10 (43.5%)	63 (67.7%)	57 (50.9%)	103 (72.5%)	40 (80.0%)	20 (87.0%)
Unknown	2 (4.3%)	6 (3.7%)	11 (7.0%)	5 (5.8%)	3 (13.0%)	0 (0.0%)	3 (2.7%)	0 (0.0%)	0 (0.0%)	0 (0.0%)
Age										
<18	0 (0.0%)	6 (3.7%)	4 (2.5%)	1 (1.2%)	0 (0.0%)	2 (2.2%)	0 (0.0%)	3 (2.1%)	0 (0.0%)	0 (0.0%)
18-64	19 (40.4%)	47 (28.7%)	67 (42.4%)	38 (44.2%)	5 (21.7%)	46 (49.5%)	52 (46.4%)	75 (52.8%)	39 (78.0%)	14 (60.9%)
65-84	22 (46.8%)	79 (48.2%)	64 (40.5%)	36 (41.9%)	10 (43.5%)	45 (48.4%)	51 (45.5%)	64 (45.1%)	11 (22.0%)	9 (39.1%)
>85	1 (2.1%)	1 (0.6%)	0 (0.0%)	0 (0.0%)	0 (0.0%)	0 (0.0%)	1 (0.9%)	0 (0.0%)	0 (0.0%)	0 (0.0%)
Unknown	5 (10.6%)	31 (18.9%)	23 (14.6%)	11 (12.8%)	8 (34.8%)	0 (0.0%)	8 (7.1%)	0 (0.0%)	0 (0.0%)	0 (0.0%)
Reporters										
HP	8 (17.0%)	32 (19.5%)	47 (29.7%)	9 (10.5%)	2 (8.7%)	2 (2.2%)	6 (5.4%)	2 (1.4%)	5 (10.0%)	1 (4.3%)
MD	29 (61.7%)	97 (59.1%)	60 (38.0%)	70 (81.4%)	20 (87.0%)	83 (89.2%)	99 (88.4%)	127 (89.4%)	42 (84.0%)	22 (95.7%)
PH	4 (8.5%)	20 (6.1%)	10 (6.3%)	5 (5.8%)	1 (4.3%)	8 (8.6%)	7 (6.3%)	13 (9.2%)	3 (6.0%)	0 (0.0%)
OT	3 (6.4%)	2 (1.2%)	25 (15.8%)	1 (1.2%)	0 (0.0%)	0 (0.0%)	0 (0.0%)	0 (0.0%)	0 (0.0%)	0 (0.0%)
CN	3 (6.4%)	23 (14.0%)	16 (10.1%)	1 (1.2%)	0 (0.0%)	0 (0.0%)	0 (0.0%)	0 (0.0%)	0 (0.0%)	0 (0.0%)
Outcomes										
Death	6 (12.8%)	45 (27.4%)	23 (14.6%)	15 (17.4%)	8 (34.8%)	0 (0.0%)	7 (6.3%)	5 (3.5%)	4 (8.0%)	2 (8.7%)
Non-death	38 (80.9%)	108 (65.9%)	128 (81.0%)	65 (75.6%)	14 (60.9%)	77 (82.8%)	84 (75.0%)	112 (78.9%)	39 (78.0%)	14 (60.9%)
Unknown	3 (6.4%)	11 (6.7%)	7 (4.4%)	6 (7.0%)	1 (4.3%)	16 (17.2%)	21 (18.8%)	25 (17.6%)	7 (14.0%)	7 (30.4%)
Reporting Year										
2012	1 (2.1%)	0 (0.0%)	0 (0.0%)	0 (0.0%)	0 (0.0%)	0 (0.0%)	0 (0.0%)	0 (0.0%)	0 (0.0%)	0 (0.0%)
2013	0 (0.0%)	0 (0.0%)	0 (0.0%)	0 (0.0%)	0 (0.0%)	0 (0.0%)	0 (0.0%)	0 (0.0%)	0 (0.0%)	0 (0.0%)
2014	0 (0.0%)	0 (0.0%)	0 (0.0%)	0 (0.0%)	0 (0.0%)	0 (0.0%)	0 (0.0%)	0 (0.0%)	0 (0.0%)	0 (0.0%)
2015	1 (2.1%)	0 (0.0%)	0 (0.0%)	0 (0.0%)	0 (0.0%)	0 (0.0%)	0 (0.0%)	0 (0.0%)	0 (0.0%)	0 (0.0%)
2016	0 (0.0%)	3 (1.8%)	1 (0.6%)	0 (0.0%)	0 (0.0%)	0 (0.0%)	0 (0.0%)	3 (2.1%)	0 (0.0%)	0 (0.0%)
2017	1 (2.1%)	1 (0.6%)	12 (7.6%)	0 (0.0%)	1 (4.3%)	0 (0.0%)	4 (3.6%)	4 (2.8%)	0 (0.0%)	0 (0.0%)
2018	0 (0.0%)	8 (4.9%)	21 (13.3%)	3 (3.5%)	2 (8.7%)	2 (2.2%)	6 (5.4%)	9 (6.3%)	2 (4.0%)	2 (8.7%)
2019	5 (10.6%)	7 (4.3%)	13 (8.2%)	13 (15.1%)	2 (8.7%)	2 (2.2%)	6 (5.4%)	9 (6.3%)	6 (12.0%)	0 (0.0%)
2020	5 (10.6%)	14 (8.5%)	21 (13.3%)	8 (9.3%)	4 (17.4%)	12 (12.9%)	8 (7.1%)	16 (11.3%)	6 (12.0%)	3 (13.0%)
2021	8 (17.0%)	19 (11.6%)	25 (15.8%)	12 (14.0%)	3 (13.0%)	15 (16.1%)	17 (15.2%)	19 (13.4%)	16 (32.0%)	1 (4.3%)
2022	7 (14.9%)	22 (13.4%)	21 (13.3%)	11 (12.8%)	3 (13.0%)	23 (24.7%)	15 (13.4%)	30 (21.1%)	12 (24.0%)	2 (8.7%)
2023	10 (21.3%)	35 (21.3%)	21 (13.3%)	18 (20.9%)	4 (17.4%)	28 (30.1%)	47 (42.0%)	39 (27.5%)	7 (14.0%)	8 (34.8%)
2024	9 (19.1%)	55 (33.5%)	23 (14.6%)	21 (24.4%)	4 (17.4%)	11 (11.8%)	9 (8.0%)	13 (9.2%)	0 (0.0%)	7 (30.4%)

IPI, Ipilimumab; PEM, Pembrolizumab; NIV, Nivolumab; ATE, Atezolizumab; DUR, Durvalumab; HP, Health-professional; MD, Physician; PH, Pharmacist; OT, Other health-professional; CN, Consumer.

ICI-related AIE was found to be more prevalent in males in both FAERS (52.3%) and JADER (67.4%). From the FAERS database, most cases are concentrated within the 65 to 84 age range, accounting for 44.1%. Conversely, from the JADER database, the predominant age group for cases is between 18 and 64 years, comprising 53.8%. Physicians constitute most reporters in both databases, with the JADER database exclusively composed of medical professionals as reporters. Within the FAERS database, 20.3% of AIE associated with ICIs resulted in fatalities, whereas this figure is significantly lower in the JADER database, at 4.3%. Notably, durvalumab is linked to the highest proportion of AIE-related deaths, accounting for 34.8% in the FAERS database and 8.7% in the JADER database.

### 3.3 NAEs among ICI-treated patients in FAERS from 2004Q1 to 2024Q4

Using the FAERS database, we examined the incidence of NAEs in patients treated with ICIs between 2004Q1 and 2024Q4. A total of 19,348,490 AE reports were included in the FAERS database. 16,564,754 reports remained after exclusion of duplicate reports, and 3,999 cases of ICI-associated NAEs were identified. Neurologic AEs accounted for only 2.35% of the adverse reactions reported in all ICIs. Recently, the number of reported cases of ICI-associated NAEs has increased annually and has attracted widespread attention. **Table 3** lists the reported annual cases of NAEs for all target drugs. The correlation analysis indicated a significant positive statistical relationship between the number of annually reported ICI-related NAE cases and the year (r = 0.9853, p < 0.0001). This finding suggests that the incidence of NAEs has progressively increased in tandem with the expanding utilization of ICIs.

**Table 3:** Reported annual cases of NAEs for all targeted drugs.

Year	NAEs	Other AEs	Total
2007	0	2	2
2008	0	5	5
2009	1	9	10
2010	0	2	2
2011	4	284	288
2012	6	1052	1058
2013	3	989	992
2014	15	1794	1809
2015	49	4490	4539
2016	137	9240	9377
2017	219	13080	13299
2018	303	15600	15903
2019	438	18397	18835
2020	463	17482	17945
2021	538	18552	19090
2022	493	21236	21729
2023	591	20589	21180
2024	739	23251	23990
Total	3999	166054	170053

NAEs, neurological immune-related adverse events; AEs, adverse events.

**Figure 2:**
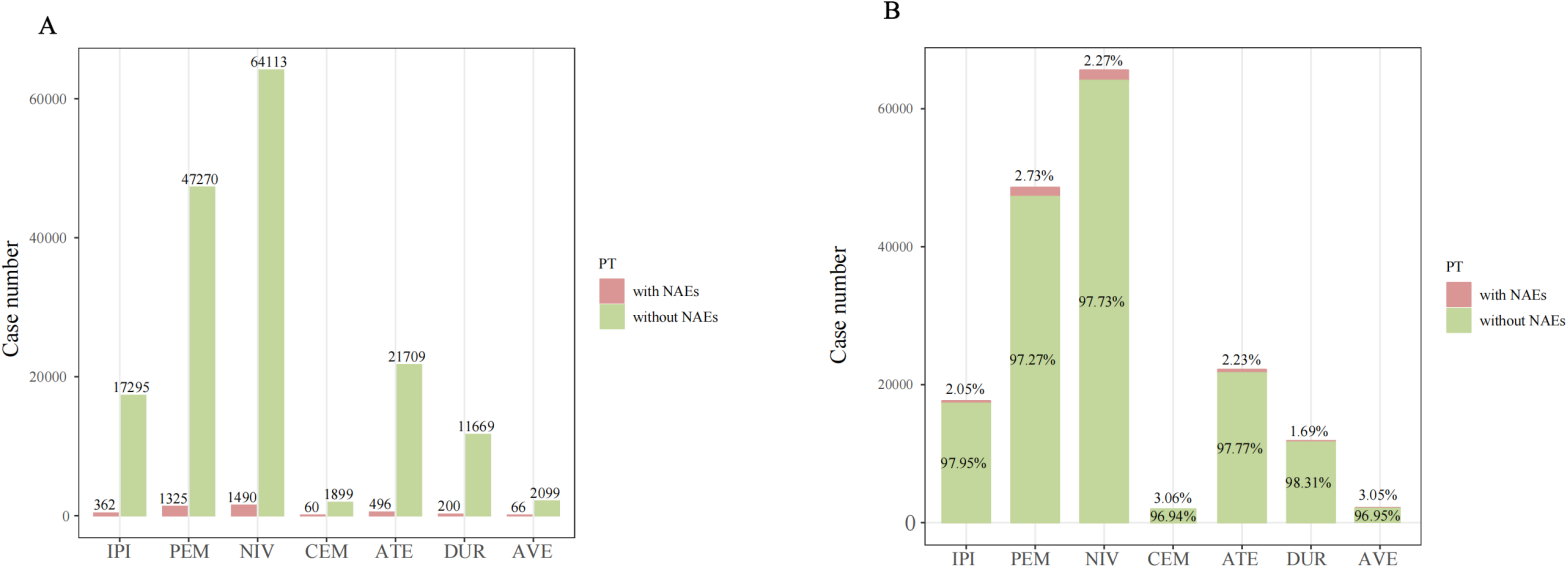
The case number **(A)** and percentage **(B)** of adverse event reports with NAEs and without NAEs of targeted ICIs in the FAERS database from 2004 Q1 to 2024 Q4. PT, preferred term; NAE, neurological immune-related adverse events.

As shown in **Figure 2**, the numbers of cases of NAEs associated with each ICI were nivolumab (N=1490), pembrolizumab (N=1325), atezolizumab (N=496), ipilimumab (N=362), durvalumab (N=200), avelumab (N=66), and cemiplimab (N=60). Although having the lowest number of cases with NAEs, cemiplimab [3.06% (60/1959)] and avelumab [3.05% (66/2165)] had the highest proportion of NAEs among all AEs.

### 3.4 Demographic characteristics of ICI-related NAEs

**Table 4** demonstrates the demographic characteristics of NAEs correlated with ICI treatment. Among all ICI-related NAEs reported, males (59.1%) were at higher risk than females (32.0%). Gender information was not available for 9.0% of cases (359/3,999). The proportion of males treated with IPI, PEM, NIV, CEM, ATE, DUR, and AVE was 63.5% (230/362), 59.6% (790/1,325), 61.3% (913/1,490), 35.0% (21/60), 50.0% (248/496), 56.0% (112/200), and 72.7% (48/66). At the same time, the proportion of females was 28.5% (103/362), 36.7% (486/1325), 30.3% (451/1490), 13.3% (8/60), 32.9% (163/496), 25.5% (51/200), and 24.2% (16/66), respectively. The age of the reported cases was predominantly 65–85 years [48.5% (1,939/3,999)], followed by 18–64 years [26.1% (1,043/3,999)], >85 years [2.3% (91/3,999)], and <18 years [1.6% (62/3,999)]. The age of the remaining cases was unknown [21.6% (864/3,999)]. An analysis of the indications for ICI treatment reveals that the primary tumors with the highest prevalence, in descending order, are lung cancer [25.9% (1034/3,999)], malignant melanoma [23.6% (944/3,999)], renal cancer [11.8% (472/3,999)], hepatic cancer [4.2% (166/3,999)], and breast cancer [2.7% (106/3,999)]. The highest percentage of reported staff types was physician [53.2% (2,127/3,999)], followed by health professional [18.6% (745/3,999)]. Among all the NAEs related to ICIs, the outcome “hospitalization” is the most frequent [35.7%, (1,429/3,999)], and the death rate is 23.7% (946/3,999). Furthermore, cemiplimab was associated with the highest mortality rate among ICIs, at 31.7% (19/60). The top 2 highest rankings among all reporting countries were the United States [29.7% (1,188/3,999)] and Japan [27.6% (1,105/3,999)].

**Table 4:** Demographic characteristics of NAEs correlated with ICIs in the FAERS database.

Characteristics	IPI	PEM	NIV	CEM	ATE	DUR	AVE
Case number	N=362	N=1325	N=1490	N=60	N=496	N=200	N=66
Gender							
Female	103 (28.5%)	486 (36.7%)	451 (30.3%)	8 (13.3%)	163 (32.9%)	51 (25.5%)	16 (24.2%)
Male	230 (63.5%)	790 (59.6%)	913 (61.3%)	21 (35.0%)	248 (50.0%)	112 (56.0%)	48 (72.7%)
Unknown	29 (8.0%)	49 (3.7%)	126 (8.5%)	31 (51.7%)	85 (17.1%)	37 (18.5%)	2 (3.0%)
Age							
<18	6 (1.7%)	25 (1.9%)	20 (1.3%)	0 (0%)	11 (2.2%)	0 (0%)	0 (0%)
18-64	129 (35.6%)	306 (23.1%)	442 (29.7%)	2 (3.3%)	127 (25.6%)	27 (13.5%)	10 (15.2%)
65-84	162 (44.8%)	687 (51.8%)	725 (48.7%)	18 (30.0%)	210 (42.3%)	92 (46.0%)	45 (68.2%)
>85	5 (1.4%)	43 (3.2%)	26 (1.7%)	2 (3.3%)	8 (1.6%)	2 (1.0%)	5 (7.6%)
Unknown	60 (16.6%)	264 (19.9%)	277 (18.6%)	38 (63.3%)	140 (28.2%)	79 (39.5%)	6 (9.1%)
Weight							
<50 kg	12 (3.3%)	37 (2.8%)	33 (2.2%)	0 (0%)	30 (6.0%)	7 (3.5%)	1 (1.5%)
50∼100 kg	101 (27.9%)	324 (24.5%)	435 (29.2%)	2 (3.3%)	165 (33.3%)	57 (28.5%)	28 (42.4%)
>100 kg	16 (4.4%)	33 (2.5%)	52 (3.5%)	1 (1.7%)	13 (2.6%)	6 (3.0%)	1 (1.5%)
Unknown	233 (64.4%)	931 (70.3%)	970 (65.1%)	57 (95.0%)	288 (58.1%)	130 (65.0%)	36 (54.5%)
Reporters							
HP	43 (11.9%)	230 (17.4%)	353 (23.7%)	8 (13.3%)	82 (16.5%)	26 (13.0%)	3 (4.5%)
MD	206 (56.9%)	646 (48.8%)	672 (45.1%)	43 (71.7%)	369 (74.4%)	139 (69.5%)	52 (78.8%)
PH	26 (7.2%)	91 (6.9%)	98 (6.6%)	3 (5.0%)	30 (6.0%)	12 (6.0%)	6 (9.1%)
OT	56 (15.5%)	59 (4.5%)	233 (15.6%)	1 (1.7%)	3 (0.6%)	6 (3.0%)	2 (3.0%)
CN	31 (8.6%)	295 (22.3%)	131 (8.8%)	5 (8.3%)	10 (2.0%)	7 (3.5%)	2 (3.0%)
Unknown	0 (0%)	4 (0.3%)	3 (0.2%)	0 (0.0%)	2 (0.4%)	10 (5.0%)	1 (1.5%)
Outcomes							
Death	61 (16.9%)	325 (24.5%)	365 (24.5%)	19 (31.7%)	106 (21.4%)	57 (28.5%)	13 (19.7%)
Life-threatening	61 (16.9%)	170 (12.8%)	249 (16.7%)	10 (16.7%)	29 (5.8%)	33 (16.5%)	4 (6.1%)
Disability	3 (0.8%)	21 (1.6%)	9 (0.6%)	1 (1.7%)	5 (1.0%)	3 (1.5%)	1 (1.5%)
Hospitalization	140 (38.7%)	485 (36.6%)	485 (32.6%)	18 (30.0%)	202 (40.7%)	65 (32.5%)	34 (51.5%)
Required intervention	0 (0%)	3 (0.2%)	0 (0%)	0 (0%)	0 (0%)	0 (0%)	0 (0%)
Congenital anomaly	0 (0%)	0 (0%)	0 (0%)	0 (0%)	0 (0%)	0 (0%)	0 (0%)
Other outcomes	87 (24.0%)	241 (18.2%)	327 (21.9%)	7 (11.7%)	117 (23.6%)	31 (15.5%)	12 (18.2%)
Unknown	10 (2.8%)	80 (6.0%)	55 (3.7%)	5 (8.3%)	37 (7.5%)	11 (5.5%)	2 (3.0%)
Reporting Country							
Japan	147 (40.6%)	326 (24.6%)	351 (23.6%)	2 (3.3%)	197 (39.7%)	65 (32.5%)	17 (25.8%)
The U.S.	100 (27.6%)	431 (32.5%)	504 (33.8%)	25 (41.7%)	81 (16.3%)	39 (19.5%)	8 (12.1%)
France	29 (8.0%)	116 (8.8%)	135 (9.1%)	7 (11.7%)	35 (7.1%)	20 (10.0%)	10 (15.2%)
Germany	22 (6.1%)	46 (3.5%)	90 (6.0%)	5 (8.3%)	19 (3.8%)	4 (2.0%)	4 (6.1%)
The UK	12 (3.3%)	47 (3.5%)	47 (3.2%)	2 (3.3%)	6 (1.2%)	11 (5.5%)	5 (7.6%)
Switzerland	6 (1.7%)	21 (1.6%)	13 (0.9%)	0 (0.0%)	5 (1.0%)	1 (0.5%)	2 (3.0%)
Austria	4 (1.1%)	6 (0.5%)	11 (0.7%)	0 (0.0%)	4 (0.8%)	1 (0.5%)	1 (1.5%)
Italy	4 (1.1%)	39 (2.9%)	48 (3.2%)	8 (13.3%)	7 (1.4%)	3 (1.5%)	2 (3.0%)
Spain	4 (1.1%)	31 (2.3%)	36 (2.4%)	1 (1.7%)	35 (7.1%)	10 (5.0%)	3 (4.5%)
Other countries	21 (6.3%)	262 (19.8%)	255 (17.1%)	10 (16.7%)	103 (20.1%)	46 (23.0%)	14 (21.2%)
Unknown	13 (3.6%)	0 (0.0%)	0 (0.0%)	0 (0.0%)	4 (0.8%)	0 (0.0%)	0 (0.0%)

HP, health-professional; MD, physician; PH, pharmacist; OT, other health-professional; CN, consumer.

### 3.5 Disproportionality analysis for ICI-related NAEs

The signal values and correlations between ICIs and NAEs are presented in **Table 5**. All drugs showed significant signals: IPI (N = 362, ROR = 5.69, 95% CI [5.16~6.28], PRR= 5.65, χ2 = 1,553.26), PEM (N = 1,325, ROR = 6.79, 95% CI [6.46~7.14], PRR= 6.73, χ2 = 7,378.53), NIV (N = 1,490, ROR = 6.21, 95% CI [5.91~6.51], PRR= 6.15, χ2 = 7,266.2), CEM (N = 60, ROR = 8.07, 95% CI [6.38~10.2], PRR= 7.98, χ2 = 433.56), ATE (N = 496, ROR = 6.43, 95% CI [5.91~6.98], PRR= 6.37, χ2 = 2,546.32), DUR (N = 200, ROR = 5.61, 95% CI [4.92~6.4], PRR= 5.57, χ2 = 850.54), AVE (N = 66, ROR = 10.4, 95% CI [8.28~13.07], PRR= 10.25, χ2 = 626.35). Notably, avelumab showed the strongest association with the nervous system compared to other ICIs. In contrast, durvalumab showed few concerns about safety of the nervous system.

**Table 5:** Safety adverse events among different ICI agents.

Drug	ICI-associated AEs n	ICI-associated NAEs n	ICI-associated NAEs as PS n	ROR (95%CI)	PRR (χ2)	EBGM (EBGM05)	IC (IC025)
IPI	44,477	407	362	5.69 (5.16-6.28)	5.65 (1,553.26)	5.63 (5.19)	2.49 (2.35)
PEM	142,628	1,537	1,325	6.79 (6.46-7.14)	6.73 (7,378.53)	6.63 (6.35)	2.73 (2.65)
NIV	174,179	1,714	1,490	6.21 (5.91-6.51)	6.15 (7,266.2)	6.05 (5.82)	2.6 (2.53)
CEM	5,480	71	60	8.07 (6.38-10.2)	7.98 (433.56)	7.97 (6.55)	2.99 (2.65)
ATE	54,848	565	496	6.43 (5.91-6.98)	6.37 (2,546.32)	6.34 (5.91)	2.66 (2.54)
DUR	25,120	227	200	5.61 (4.92-6.4)	5.57 (850.54)	5.56 (4.98)	2.47 (2.28)
AVE	4,506	75	66	10.4 (8.28-13.07)	10.25 (626.35)	10.24 (8.46)	3.36 (3.02)

PS, primary suspect; ROR, reporting odds ratio; PRR, proportional reporting ratio; χ2, chi-squared; EBGM, empirical Bayesian geometric mean; EBGM05, the lower limit of 95% CI of EBGM; IC, information component; IC025, the lower limit of 95% CI of the IC.

The PT signals of all ICI-associated NAEs are listed in the **Supplementary Table S2**. Noninfectious myelitis, acute disseminated encephalomyelitis, and multiple sclerosis had no positive signal in all 4 algorithms. **Supplementary Table S3** displays the signal strength of PTs associated with each ICI agent. The signal ranges of ROR values for each drug were: IPI (0.19~203.14, median 11.94), PEM (0.17~284.55, median 9.66), NIV (0.13~177.49, median 9.02), CEM (2.34~137.59, median 23.895), ATE (0.09~104.26, median 11.195), DUR (0.05~102.83, median 14.515), and AVE (3.05~140.03, median 35.625).

**Figure 3:**
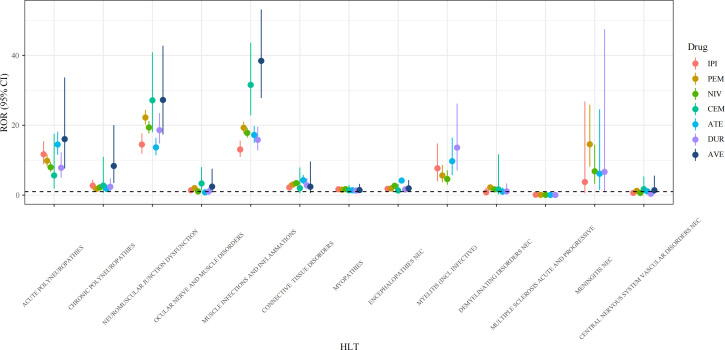
Forest plots of ROR values under HLT levels of different ICI agents. HLT, high level term; ROR, reporting odds ratio; CI, confidence interval; IPI, ipilimumab; PEM, pembrolizumab; NIV, nivolumab; CEM, cemiplimab; ATE, atezolizumab; DUR, durvalumab; AVE, avelumab.

Using high-level terms (HLT), AEs at the PT level were clustered into subsequent classifications according to neurological diseases. As shown in **Figure 3**, at the HLT level, the signal ranges of ROR values for the 7 drugs were: IPI (0.11~14.48, median 2.2), PEM (0.09~22.21, median 2.22), NIV (0.07~19.37, median 2.68), CEM (1.33~31.55, median 2.36), ATE (0.05~17.21, median 4.18), DUR (0.04~18.6, median 2.39), and AVE (1.4~38.43, median 2.44).

**Figure 4:**
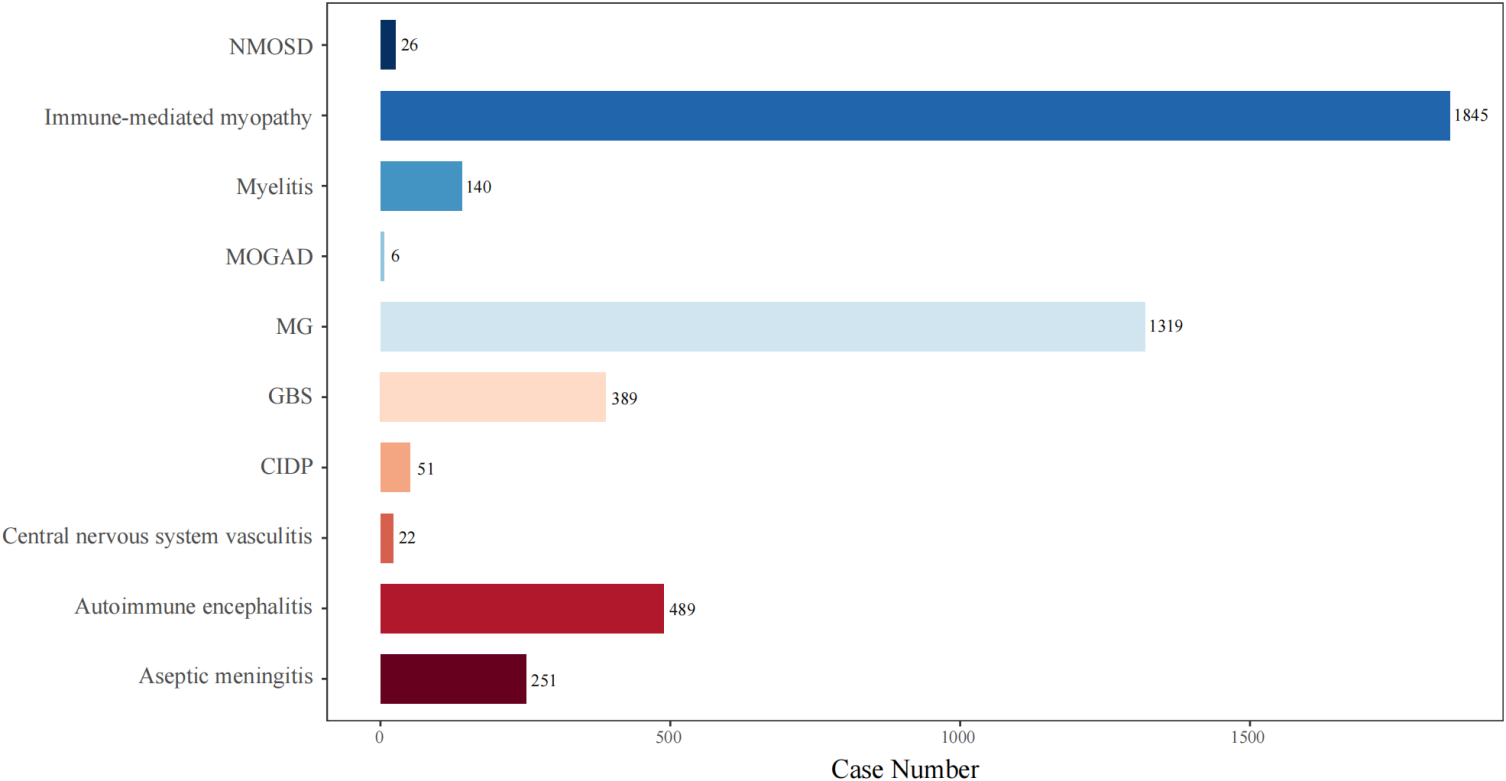
The number of reported cases of the top 10 types of NAEs associated with ICI therapies in the FAERS database. NMOSD, neuromyelitis spectrum disorder; MOGAD, myelin-oligodendrocyte glycoprotein antibody-associated disease; MG, myasthenia gravis; GBS, Guillain-Barré syndrome; CIDP, chronic inflammatory demyelinating polyradiculoneuropathy.

To better understand the clinical features of NAEs, we combined the names of PTs representing the same disease identity. We also explored the top 10 most frequently reported NAEs following ICI treatment. As shown in **Figure 4**, the NAEs were: immune-mediated myopathy (N=1845), MG (N=1319), AIE (N=489), GBS (N=389), aseptic meningitis (N=251), myelitis (N=140), CIDP (N=51), NMOSD (N=26), CNS vasculitis (N=22), and MOGAD (N=6).

**Figure 5:**
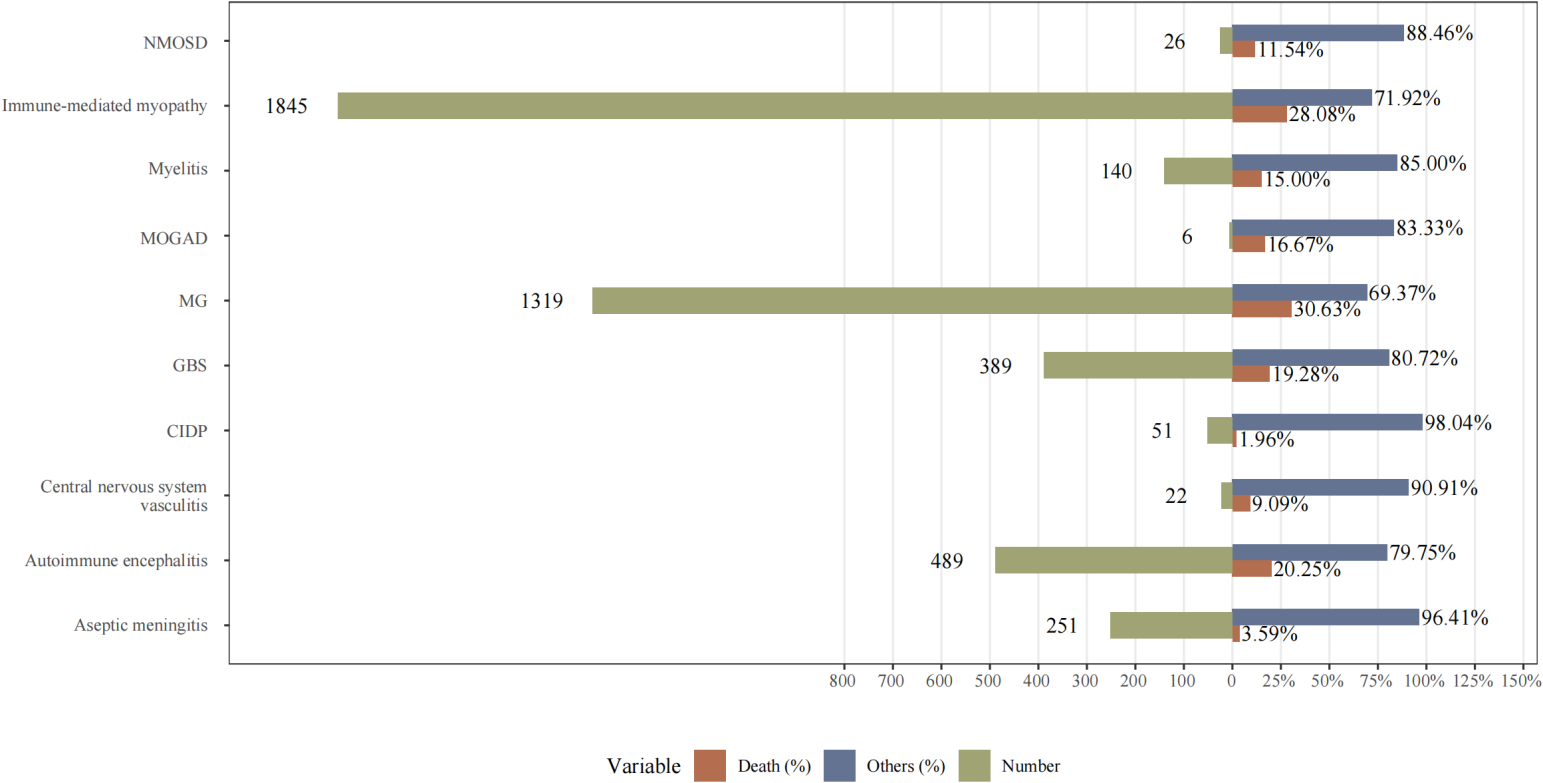
The mortality rates (number of reported deaths/number of reported adverse events) for various NAEs following ICI treatment. NMOSD, neuromyelitis spectrum disorder; MOGAD, myelin-oligodendrocyte glycoprotein antibody-associated disease; MG, myasthenia gravis; GBS, Guillain-Barré syndrome; CIDP, chronic inflammatory demyelinating polyradiculoneuropathy.

As shown in **Figure 5**, we calculated the mortality rates (number of reported deaths/number of reported AEs) for various NAEs following ICI treatment. Importantly, MG exhibited the highest mortality rate at 30.63%, followed by immune-mediated myopathy at 28.08%, and AIE at 20.25%. Therefore, these findings underscore the importance of early recognition and proactive immunotherapeutic intervention by clinicians.

### 3.6 Time to onset of neurological adverse event

**Figure 6:**
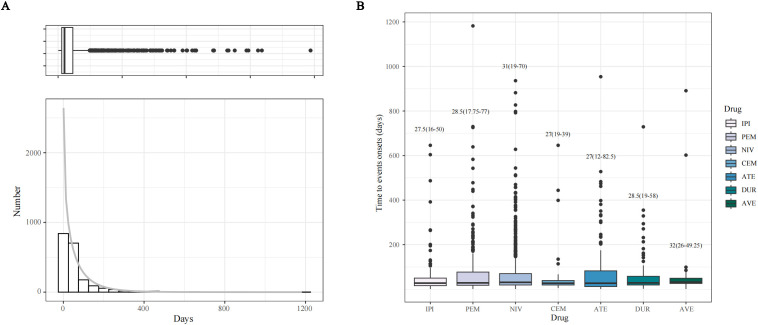
Time to onset (TTO) analysis (counted in days) of ICI-related NAEs. **(A)** Histogram displaying the adverse event numbers corresponding to TTO of ICI-related NAEs. **(B)** Boxplot of the TTO of NAEs for each ICI agents.

**Figure 6a** presents a histogram showing the incidence of NAEs during ICI treatment. **Figure 6b** depicts the duration of NAE episodes induced by each ICI drug. The median onset time of NAEs associated with all target drugs was 30 days (interquartile range [IQR]: 17–69 days). Additionally, the median time to onset of NAEs was earliest in the CEM and ATE groups, at 21 days (IQR: 10 to 39 days in the CEM group and IQR: 12 to 82.5 days in the ATE group). Notably, the ATE group had the widest range of time to onset. The median time to onset of NAEs in the AVE group was the longest at 32 days (IQR: 26 to 49.25 days). The median times to onset of NAEs for IPI, PEM, NIV and DUR were 27.5 days (IQR: 16–50 days), 28.5 days (IQR: 17.75–77 days), 31 days (IQR: 19–70 days) and 28.5 days (IQR: 19–58 days), respectively. Thus, the investigation into the onset time of NAEs induced by ICIs provides novel insights into the clinical application of these drugs and the early identification of neurological toxicity.

### 3.7 Comparison between serious and non-serious groups for ICI-related NAEs

As presented in **Table 6**, the following NAE types correlated with the severity of case outcome with a p-value <0.05: aseptic meningitis (χ2 = 64.89, p<0.001), AIE (χ2 = 6.55, p=0.01), CIDP (χ2 = 14.58, p<0.001), GBS (χ2 = 7.37, p=0.01), MG (χ2 = 31.69, p<0.001), myelitis (χ2 = 7.04, p=0.01), immune-related myopathy (χ2 = 15.92, <0.001). In contrast, there was no significant correlation between other NAE types and the severity of the outcome: CNS vasculitis (χ2 = 2.98, p=0.14), and myelin oligodendrocyte glycoprotein antibody-associated disease (χ2 = 0.22, p=1).

**Table 6:** Comparison between the serious and non-serious groups for ICI-related NAEs.

PT, N (%)	Serious cases	Non-serious cases	χ2	p-value
Aseptic meningitis	9 (0.79%)	242 (7.10%)	64.89	<0.001
Autoimmune encephalitis	99 (8.70%)	390 (11.00%)	6.55	0.01
Central nervous system vasculitis	2 (0.18%)	20 (0.59%)	2.98	0.14
Chronic inflammatory demyelinating polyradiculoneuropathy	1 (0.09%)	50 (1.50%)	14.58	<0.001
Guillain-Barre syndrome	75 (6.60%)	314 (9.20%)	7.37	0.01
Myasthenia gravis	404 (36.00%)	915 (27.00%)	31.69	<0.001
Myelin oligodendrocyte glycoprotein antibody-associated disease	1 (0.09%)	5 (0.15%)	0.22	1
Myelitis	22 (1.90%)	120 (3.50%)	7.04	0.01
Immune-mediated myopathy	518 (46.00%)	1327 (39.00%)	15.92	<0.001
Neuromyelitis optica spectrum disorder	3 (0.26%)	23 (0.68%)	2.52	0.17

## 4 Discussion

ICI therapies have been revolutionizing the treatment of malignant neoplasms. Characterizing neurological immunotoxicity associated with emerging cancer immunotherapies constitutes a new and growing area of research. As such, the prevalence of NAEs is expected to increase with the expansion of ICI indications. Currently, there is no unified diagnostic standard for immunotherapy-related neurological toxicity, which remains a diagnosis of exclusion that requires differentiation from infectious diseases, metabolic disorders, and tumor metastasis.

In this study, data from the FAERS database suggest a higher likelihood of NAEs following ICI therapy in males compared to females, aligning with prior systematic reviews (15). The median onset time for ICI-induced NAEs was 30 days (IQR: 17~69 days). Prior findings from institutions like the Mayo Clinic and the Royal Marsden Hospital suggested a median onset time for NAEs of around 3 treatment cycles (16, 17). Furthermore, delayed immune-related events (DIRE) were noted, with a median interval of 6 months after immunotherapy (18). The longest time until the onset of AEs in this research was 1182 days, observed in a patient undergoing pembrolizumab treatment for malignant melanoma.

AIE is the most common CNS irAE. Manifestations of ICI-induced AIE may include headache, altered mental status, cognitive deficits, seizures, ataxia, dysphagia, and aphasia. Approximately 50% of patients exhibit positive neuronal antibodies, most commonly against intracellular antigens (especially Ma2 or Hu), the presence of which usually indicates a poor prognosis (19, 20). Patients with positive anti-glutamic acid decarboxylase or anti-neuronal surface antigen antibodies respond to treatment well and tend to have a favorable prognosis. Neuropathologic features include diffuse, cytotoxic CD8+ T cell infiltration, reactive astrogliosis, and activation of microglial cells (10).

Approximately two-thirds of ICI-induced NAEs involve the PNS. Consistent with prior studies, myopathy is the most prevalent ICI-mediated NAE. Unlike idiopathic inflammatory myopathy, immune-related myopathy predominantly involves extraocular and bulbar muscles. It can also coexist with MG and myocarditis (21). Immune-related myopathy typically manifests within the first 4 weeks of ICI therapy, often following the initial or second treatment cycle (22, 23). Patients with overlap syndrome exhibit more severe symptoms, such as ptosis, bulbar dysfunction, and dyspnea, compared to those with myositis alone. Notably, most patients display elevated creatine phosphokinase (CPK) levels, exceeding more than twice the upper limit of the normal range. Some cases test positive for myositis-specific antibodies, myositis-associated antibodies, and anti-striated muscle antibodies (AsM-Ab) (24). Approximately 5% of patients with immune-related myopathy present with a dermatomyositis-like rash, and those with this rash often have high titers of autoantibodies against TIF1γ (25). In most instances, muscle biopsies commonly exhibit notable necrosis, macrophagy, and muscle regeneration, along with perivascular inflammatory infiltrates marked by a significant abundance of macrophagic cells (26). In prior research, transcriptomic analysis of muscle biopsies has revealed 3 distinct types of ICI-myositis. The IL-6 pathway was overexpressed across all groups. Type I interferon pathway activation was unique to ICI-DM (dermatomyositis). In contrast, the type 2 IFN pathway was overexpressed in ICI-DM and ICI-MYO1. Myocarditis was observed exclusively in ICI-MYO1 patients (23).

MG is an autoimmune disorder characterized by impaired neuromuscular transmission due to autoantibodies targeting postsynaptic membrane components at the neuromuscular junction. A retrospective clinical study on ICI-induced MG reported a serum anti-acetylcholine receptor (AChR) antibody positivity rate of approximately 66% and an AsM-Ab positivity rate of about 67%. A noteworthy observation is that a small subset of patients demonstrated anti-AChR antibodies before initiating ICI treatment, with antibody titers increasing by at least twofold following treatment. Elevated serum CPK levels were also observed in 84% of the patients, and those with elevated CPK levels were more likely to experience respiratory failure. Overall, the mortality rate among patients with ICI-related MG was approximately 37%, with 15% succumbing to complications related to MG (27).

The precise mechanism underlying NAEs remains poorly understood; however, several contributing factors have been identified (1): ICIs may deplete regulatory T cells (Tregs), leading to a loss of immune tolerance (28); (2) ICIs can elevate the titers of preexisting autoantibodies that target normal nervous system tissues, and may also enhance cross-reactivity between antigens shared by tumor cells and normal neurons (29); (3) pro-inflammatory factors such as C-X-C motif chemokine ligand (CXCL)-10, C-C motif chemokine ligand (CCL)-3, CCL4, CCL5, and tumor necrosis factor (TNF) have been found elevated in prior studies (30). (4) The activation of the complement system may further promote an inflammatory response (31). (5) Specific gut microbiota modulate immune and nervous system functions, thereby influencing the pathophysiological processes associated with neuroinflammation and neural injury (32, 33). Given that potential pathogenic mechanisms underlying NAEs may involve shared antigens between tumors and normal tissues, there may be a possible association between the occurrence of NAEs and the type of primary tumor. In this study, the tumor types most frequently associated with NAEs following ICI treatment include lung cancer, malignant melanoma, renal cancer, hepatic cancer, and breast cancer.

According to Common Terminology Criteria for AEs, for irAEs exceeding Grade 2, glucocorticoids are the first-line therapy for NAEs. Additionally, the high-dose pulse therapy is usually applied to control symptoms rapidly. For those who do not respond well to glucocorticoids, it may be necessary to consider using other agents such as intravenous immunoglobulin (IVIG), immunosuppressants, rituximab, TNF-α antibodies, and interleukin-6 receptor inhibitors. Patients experiencing Grade ≥3 irAEs need to discontinue ICIs permanently (34–36). Immune-related markers, including peripheral blood lymphocyte subsets and pro-inflammatory factors, in patients undergoing ICI therapy may aid in predicting the onset of NAEs. Nonetheless, effective preventive strategies remain insufficient, and the prophylactic administration of medications such as glucocorticoids is discouraged due to potential complications, including severe infections. Despite these challenges, achieving an early and accurate diagnosis following the emergence of NAEs and administering treatment tailored to the severity of the condition can enhance patient prognosis. Thus, engaging a multidisciplinary team in decision-making is essential for patients undergoing ICI treatment. Furthermore, discontinuation of tumor therapy may compromise treatment effectiveness, whereas severe neurological irAEs could potentially impact patient survival.

Previous research indicated that the occurrence of irAEs may be associated with the therapeutic efficacy of ICIs. Patients who experience irAEs exhibit a 23-fold higher probability of clinical response than those without irAEs, suggesting that irAEs might indicate an immune response against tumors. Correspondingly, patients with effective tumor regression are more likely to develop autoimmune toxicity. Therefore, oncologists should remain highly vigilant for the occurrence of NAEs in patients with tumor regression following ICI treatment. However, the underlying mechanism of this association remains unclear (37).

In this study, the proportion of total death cases among all ICI-induced NAEs included was 23.7%. Death rates for different NAEs ranged from 1.96% to 30.63%, with myositis and dermatomyositis exhibiting the highest mortality rate (30.63%) and CIDP the lowest (1.96%). Moreover, we found that some PTs are associated with the severity of outcomes. Significant differences (p < 0.05) were observed between severe and non-severe cases across various NAEs, including aseptic meningitis, AIE, CIDP, GBS, MG, myelitis, and immune-related myopathy.

Similar to other spontaneous reporting systems, the FAERS and JADER databases have inherent limitations. Thus, it exclusively documents drug-related adverse reactions, rendering it inadequate for assessing the incidence of specific AEs within the general population. The databases comprise complex data sources characterized by substantial missing information, including variables such as gender, age, dosage, and method of administration. Additionally, challenges such as non-standardized reporting nomenclature, redundant reports, and non-professional reporters may introduce reporting bias. Within the FAERS and JADER, reports are not required to establish a causal link between drugs and adverse events prior to submission. The disproportionality analysis employed can only indicate a statistical association rather than establish causation between ICIs and NAEs. This study focused on data where ICIs were identified as the primary suspect (PS) agents, without accounting for the potential influence of patients’ concurrent medications and other variables such as infections. This limitation complicates the task of distinguishing signals attributable solely to ICIs from those arising from the combined effects of concurrent medications. Consequently, large-scale epidemiological studies are necessary for future validation.

## 5 Conclusion

This study has demonstrated a significant association between ICIs and certain NAEs, including AIE. While NAEs occur less frequently than irAEs affecting other organ systems, their clinical impact can be severe, leading to life-threatening complications and necessitating vigilant monitoring. These findings contribute to a deeper understanding of these complications, facilitating earlier detection and more effective management strategies for patients undergoing ICI therapy. The insights gained may help clinicians optimize treatment protocols while minimizing neurological risks associated with immunotherapy. Significantly, consensus guidelines currently rely on empirical data. Therefore, future large-scale prospective clinical studies will be essential for investigating the pathogenesis of NAEs and developing effective prevention and treatment strategies.

## Data Availability

The original contributions presented in the study are included in the article/**Supplementary Material**, further inquiries can be directed to the corresponding author/s.
