# Supplementary material for: Immune checkpoint inhibitor-associated autoimmune encephalitis and other neurological immune-mediated adverse events: a pharmacovigilance study using the FAERS and JADER
Source: Front Oncol. 2025 Jul 17;15:1621045. doi: 10.3389/fonc.2025.1621045 (PMC12310646; doi:10.3389/fonc.2025.1621045)
Supplement: Supplementary file 1 [file Table1.docx]

Supplementary Material

**Supplementary Table S1** Formulas and signal detection criteria for the four algorithms.

| **Algorithms** | **Equations** | | | **Criteria** | |  |
| --- | --- | --- | --- | --- | --- | --- |
| ROR | ROR=(a/b)/(c/d) | | | Lower limit of 95% CI>1, a≥3 | |  |
|  | 95%CI=e^ln(ROR)±1.96(1/a+1/b+1/c+1/d)^0.5^ | | |  |  |  |
| PRR | PRR=(a/(a+b))/(c/(c+d)) | | | Lower limit of 95% CI>1, a≥3 | |  |
|  | SE(lnPRR)=(1/a-1/(a+b)+1/c-1/(c+d))^0.5 | | |  |  |  |
|  | 95%CI=e^ln(PRR)±1.96(1/a-1/(a+b)+1/c-1/(c+d))^0.5^ | | |  |  |  |
| BCPNN | IC=log_2_a(a+b+c+d)/((a+b)(a+c)) | | | IC025>0 | |  |
|  | E(IC)=log2[(a+γ11)(N+α)(N+β)/((N+γ)(a+b+α1)(a+c+β1))] | | |  |  |  |
|  | V(IC)=1/(ln2)^2[(N-a+γ-γ11)/((a+γ11)(1+N+γ))+ (N-a-b+α-α1)/((a+b+α1)(1+N+α))+(N-a-c+β-β1)/((a+c+β1)(1+N+β)) ] | | |  |  |  |
|  | γ=γ11(N+α)(N+β)/((a+b+α1)(a+b+β1)) | | |  |  |  |
|  | IC-2SD=E(IC)-2(V(IC))^0.5 | | |  |  |  |
|  | α1=β1=1, α=β=2, γ11=1 | | |  |  |  |
| MGPS | EBGM=a(a+b+c+d)/((a+c)(a+b)) | | | EBGM05>2, a>0 | |  |
|  | EBGM05=e^ln(EBGM)-1.64(1/a+1/b+1/c+1/d)^0.5^ | | |  |  |  |
|  | | **Reports with the suspected adverse effect** | **Reports without the suspected adverse effect** | | **Total** | |
| Reports with the suspected drug | | a | b | | a+b | |
| Reports without the suspected drug | | c | d | | c+d | |
| Total | | a+c | b+d | | *N* = a+b+c+d | |

**Supplementary Table S2** Signal values for all targeted ICI drugs at the PT level.

| **PT** | **a** | **b** | **c** | **d** | **ROR (95%CI)** | **PRR (χ2)** | **EBGM (EBGM05)** | **IC (IC025)** |
| --- | --- | --- | --- | --- | --- | --- | --- | --- |
| Gullain-Barre syndrome | 338 | 450900 | 3892 | 53799471 | 10.36 (9.27-11.58) | 10.35 (2628.56) | 9.61 (8.75) | 3.26 (3.1) |
| Miller Fisher syndrome | 27 | 451211 | 165 | 53803198 | 19.51 (12.99-29.31) | 19.51 (407.5) | 16.91 (12.03) | 4.08 (3.5) |
| Acuter motor axonal neuropathy | 12 | 451226 | 77 | 53803286 | 18.58 (10.11-34.14) | 18.58 (172.71) | 16.21 (9.74) | 4.02 (3.16) |
| Acuter motor-sensory axonal neuropathy | 12 | 451226 | 180 | 53803183 | 7.95 (4.43-14.26) | 7.95 (68.34) | 7.51 (4.61) | 2.91 (2.08) |
| Chronic inflammatory demyelinating polyradiculoneuropathy | 51 | 451187 | 1077 | 53802286 | 5.65 (4.26-7.48) | 5.65 (186.18) | 5.44 (4.3) | 2.44 (2.03) |
| Immune-mediated myasthenia gravis | 155 | 451083 | 21 | 53803342 | 880.37 (558.12-1388.68) | 880.07 (16239.34) | 105.89 (72.31) | 6.73 (6.41) |
| Myasthenia gravis | 922 | 450316 | 5375 | 53797988 | 20.49 (19.11-21.98) | 20.45 (14562.68) | 17.6 (16.6) | 4.14 (4.04) |
| Myasthenia gravis crisis | 56 | 451182 | 1082 | 53802281 | 6.17 (4.72-8.07) | 6.17 (230.72) | 5.92 (4.73) | 2.56 (2.17) |
| Myasthenic syndrome | 153 | 451085 | 474 | 53802889 | 38.5 (32.09-46.2) | 38.49 (4223.35) | 29.34 (25.19) | 4.87 (4.62) |
| Ocular myasthenia | 33 | 451205 | 270 | 53803093 | 14.57 (10.15-20.92) | 14.57 (371.75) | 13.09 (9.68) | 3.71 (3.19) |
| Autoimmune myositis | 49 | 451189 | 243 | 53803120 | 24.05 (17.69-32.68) | 24.04 (900.57) | 20.18 (15.61) | 4.33 (3.89) |
| Dermatomyositis | 151 | 451087 | 2081 | 53801282 | 8.65 (7.34-10.21) | 8.65 (952.79) | 8.13 (7.08) | 3.02 (2.78) |
| Immune-mediated myositis | 396 | 450842 | 1422 | 53801941 | 33.23 (29.73-37.15) | 33.2 (9675.07) | 26.19 (23.86) | 4.71 (4.55) |
| Myositis | 1116 | 450122 | 6571 | 53796792 | 20.3 (19.05-21.63) | 20.25 (17460.21) | 17.46 (16.55) | 4.13 (4.03) |
| Necrotising myositis | 30 | 451208 | 626 | 53802737 | 5.71 (3.96-8.24) | 5.71 (111.34) | 5.5 (4.05) | 2.46 (1.93) |
| Polymyositis | 103 | 451135 | 1514 | 53801849 | 8.11 (6.65-9.91) | 8.11 (601.32) | 7.66 (6.48) | 2.94 (2.65) |
| Autoimmune encephalopathy | 24 | 451214 | 63 | 53803300 | 45.43 (28.39-72.69) | 45.42 (755.04) | 33.17 (22.38) | 5.05 (4.4) |
| Encephalitis autoimmune | 208 | 451030 | 803 | 53802560 | 30.9 (26.53-35.99) | 30.89 (4777.48) | 24.74 (21.77) | 4.63 (4.41) |
| Immune-mediated encephalitis | 224 | 451014 | 80 | 53803283 | 334.02 (258.76-431.17) | 333.86 (19562.44) | 88.59 (71.55) | 6.47 (6.22) |
| Noninfective encephalitis | 33 | 451205 | 578 | 53802785 | 6.81 (4.79-9.67) | 6.81 (154.67) | 6.49 (4.84) | 2.7 (2.19) |
| Immune-mediated myelitis | 5 | 451233 | 2 | 53803361 | 298.09 (57.83-1536.49) | 298.09 (422.99) | 85.88 (21.78) | 6.42 (4.87) |
| Myelitis | 71 | 451167 | 1279 | 53802084 | 6.62 (5.21-8.41) | 6.62 (320.87) | 6.32 (5.18) | 2.66 (2.31) |
| Myelitis transverse | 64 | 451174 | 1168 | 53802195 | 6.53 (5.08-8.4) | 6.53 (284.36) | 6.25 (5.06) | 2.64 (2.28) |
| Noninfectious myelitis | 2 | 451236 | 72 | 53803291 | 3.31 (0.81-13.5) | 3.31 (3.14) | 3.25 (1) | 1.7 (0) |
| Neuromyelitis optica spectrum disorder | 26 | 451212 | 895 | 53802468 | 3.46 (2.35-5.12) | 3.46 (44.28) | 3.39 (2.45) | 1.76 (1.2) |
| Acute disseminated encephalomyelitis | 5 | 451233 | 318 | 53803045 | 1.87 (0.77-4.54) | 1.87 (2.01) | 1.86 (0.89) | 0.9 (-0.29) |
| Multiple sclerosis | 51 | 451187 | 44535 | 53758828 | 0.14 (0.1-0.18) | 0.14 (278.38) | 0.14 (0.11) | -2.86 (-3.26) |
| Myelin oligodendrocyte glycoprotein antibody-associated disease | 6 | 451232 | 73 | 53803290 | 9.8 (4.26-22.53) | 9.8 (43.81) | 9.13 (4.55) | 3.19 (2.05) |
| Meningitis aseptic | 247 | 450991 | 3696 | 53799667 | 7.97 (7.01-9.07) | 7.97 (1411) | 7.53 (6.76) | 2.91 (2.72) |
| Meningitis noninfective | 4 | 451234 | 45 | 53803318 | 10.6 (3.81-29.47) | 10.6 (31.93) | 9.82 (4.17) | 3.3 (1.94) |
| Central nervous system vasculitis | 22 | 451216 | 390 | 53802973 | 6.73 (4.38-10.34) | 6.73 (101.52) | 6.42 (4.48) | 2.68 (2.06) |

**Supplementary Table S3** Signal strength of NAEs corresponding to each ICI drug respectively at the PT level.

| **Drugs** | **Preferred term (PT)** | **Report number** | **ROR (95%CI)** | **PRR (χ2)** | **EBGM (EBGM05)** | **IC (IC025)** |
| --- | --- | --- | --- | --- | --- | --- |
| Ipilimumab | Myositis | 79 | 12.68 (10.15 - 15.83) | 12.66 (839.48) | 12.54 (10.41) | 3.65 (3.32) |
|  | Myasthenia gravis | 67 | 13.13 (10.32 - 16.7) | 13.11 (741.45) | 12.98 (10.61) | 3.7 (3.35) |
|  | Meningitis aseptic | 41 | 12.82 (9.42 - 17.44) | 12.81 (441.67) | 12.68 (9.8) | 3.66 (3.22) |
|  | Guillain-Barre syndrome | 41 | 11.94 (8.78 - 16.24) | 11.93 (406.59) | 11.82 (9.14) | 3.56 (3.12) |
|  | Immune-mediated myositis | 34 | 23.25 (16.55 - 32.64) | 23.23 (709.74) | 22.81 (17.17) | 4.51 (4.02) |
|  | Immune-mediated encephalitis | 28 | 123.73 (83.87 - 182.53) | 123.65 (3092.69) | 112.35 (81.15) | 6.81 (6.25) |
|  | Dermatomyositis | 15 | 8.25 (4.96 - 13.71) | 8.25 (94.88) | 8.2 (5.36) | 3.04 (2.31) |
|  | Myasthenic syndrome | 15 | 29.88 (17.9 - 49.88) | 29.87 (408.59) | 29.18 (19.01) | 4.87 (4.14) |
|  | Encephalitis autoimmune | 14 | 17.12 (10.1 - 29.02) | 17.12 (209.49) | 16.89 (10.86) | 4.08 (3.33) |
|  | Immune-mediated myasthenia gravis | 12 | 89.21 (49.64 - 160.32) | 89.18 (974.99) | 83.17 (50.93) | 6.38 (5.55) |
|  | Myelitis | 11 | 10.02 (5.53 - 18.13) | 10.01 (88.51) | 9.94 (6.05) | 3.31 (2.48) |
|  | Myelitis transverse | 7 | 6.97 (3.31 - 14.64) | 6.96 (35.56) | 6.93 (3.72) | 2.79 (1.77) |
|  | Multiple sclerosis | 7 | 0.19 (0.09 - 0.4) | 0.19 (23.93) | 0.19 (0.1) | -2.38 (-3.4) |
|  | Chronic inflammatory demyelinating polyradiculoneuropathy | 7 | 7.61 (3.62 - 16) | 7.61 (39.95) | 7.57 (4.06) | 2.92 (1.9) |
|  | Polymyositis | 6 | 4.54 (2.04 - 10.12) | 4.54 (16.5) | 4.53 (2.31) | 2.18 (1.09) |
|  | Myasthenia gravis crisis | 5 | 5.38 (2.23 - 12.95) | 5.38 (17.75) | 5.36 (2.57) | 2.42 (1.24) |
|  | Autoimmune encephalopathy | 3 | 43.53 (13.76 - 137.71) | 43.53 (120.36) | 42.06 (16.05) | 5.39 (3.92) |
|  | Acute motor-sensory axonal neuropathy | 3 | 19.35 (6.18 - 60.53) | 19.35 (51.38) | 19.06 (7.34) | 4.25 (2.79) |
|  | Noninfective encephalitis | 2 | 4 (1 - 16.04) | 4 (4.49) | 3.99 (1.25) | 2 (0.33) |
|  | Central nervous system vasculitis | 2 | 5.95 (1.48 - 23.86) | 5.95 (8.19) | 5.92 (1.85) | 2.57 (0.89) |
|  | Autoimmune myositis | 2 | 8.41 (2.09 - 33.77) | 8.41 (12.96) | 8.36 (2.61) | 3.06 (1.39) |
|  | Noninfectious myelitis | 1 | 16.7 (2.32 - 120.13) | 16.7 (14.56) | 16.48 (3.16) | 4.04 (1.98) |
|  | Neuromyelitis optica spectrum disorder | 1 | 1.32 (0.19 - 9.42) | 1.32 (0.08) | 1.32 (0.26) | 0.41 (-1.64) |
|  | Necrotising myositis | 1 | 1.86 (0.26 - 13.23) | 1.86 (0.4) | 1.86 (0.36) | 0.89 (-1.15) |
|  | Ocular myasthenia | 1 | 4.04 (0.57 - 28.75) | 4.04 (2.28) | 4.03 (0.78) | 2.01 (-0.04) |
|  | Acute motor axonal neuropathy | 1 | 13.85 (1.93 - 99.43) | 13.85 (11.79) | 13.71 (2.63) | 3.78 (1.71) |
|  | Immune-mediated myelitis | 1 | 203.14 (24.46 - 1687.45) | 203.14 (172.41) | 174.26 (29.64) | 7.45 (5.16) |
| Pembrolizumab | Myositis | 323 | 16.68 (14.92 - 18.64) | 16.64 (4549.55) | 15.98 (14.56) | 4 (3.83) |
|  | Myasthenia gravis | 321 | 20.42 (18.25 - 22.85) | 20.38 (5614.52) | 19.39 (17.65) | 4.28 (4.11) |
|  | Immune-mediated myositis | 189 | 44.07 (37.91 - 51.24) | 44.02 (7119.88) | 39.55 (34.86) | 5.31 (5.09) |
|  | Guillain-Barre syndrome | 105 | 9.66 (7.96 - 11.73) | 9.66 (794.72) | 9.44 (8.03) | 3.24 (2.96) |
|  | Immune-mediated encephalitis | 94 | 169.93 (133.24 - 216.74) | 169.82 (10897.91) | 117.62 (95.96) | 6.88 (6.54) |
|  | Immune-mediated myasthenia gravis | 70 | 250.66 (185.34 - 339.01) | 250.54 (10478.51) | 151.29 (117.52) | 7.24 (6.84) |
|  | Meningitis aseptic | 66 | 6.46 (5.07 - 8.24) | 6.46 (299.41) | 6.37 (5.19) | 2.67 (2.32) |
|  | Encephalitis autoimmune | 54 | 21.42 (16.28 - 28.17) | 21.41 (994.45) | 20.32 (16.15) | 4.34 (3.95) |
|  | Myasthenic syndrome | 49 | 32.17 (24.03 - 43.07) | 32.16 (1363.9) | 29.73 (23.29) | 4.89 (4.47) |
|  | Dermatomyositis | 33 | 5.69 (4.04 - 8.03) | 5.69 (125.8) | 5.62 (4.22) | 2.49 (1.99) |
|  | Polymyositis | 30 | 7.17 (5 - 10.29) | 7.17 (156.39) | 7.06 (5.22) | 2.82 (2.3) |
|  | Myasthenia gravis crisis | 24 | 8.17 (5.46 - 12.25) | 8.17 (147.92) | 8.02 (5.72) | 3 (2.42) |
|  | Multiple sclerosis | 20 | 0.17 (0.11 - 0.26) | 0.17 (80.9) | 0.17 (0.12) | -2.55 (-3.18) |
|  | Autoimmune myositis | 18 | 24.93 (15.47 - 40.16) | 24.92 (387.87) | 23.45 (15.73) | 4.55 (3.87) |
|  | Myelitis transverse | 16 | 4.99 (3.05 - 8.18) | 4.99 (50.42) | 4.94 (3.27) | 2.3 (1.6) |
|  | Chronic inflammatory demyelinating polyradiculoneuropathy | 16 | 5.46 (3.33 - 8.94) | 5.46 (57.45) | 5.4 (3.57) | 2.43 (1.73) |
|  | Ocular myasthenia | 14 | 18.38 (10.75 - 31.43) | 18.38 (219.44) | 17.58 (11.22) | 4.14 (3.37) |
|  | Necrotising myositis | 13 | 7.67 (4.43 - 13.28) | 7.67 (73.92) | 7.54 (4.76) | 2.91 (2.13) |
|  | Myelitis | 13 | 3.69 (2.14 - 6.37) | 3.69 (25.24) | 3.66 (2.32) | 1.87 (1.1) |
|  | Neuromyelitis optica spectrum disorder | 12 | 5.01 (2.83 - 8.85) | 5.01 (38) | 4.96 (3.08) | 2.31 (1.5) |
|  | Noninfective encephalitis | 10 | 6.31 (3.38 - 11.79) | 6.31 (43.98) | 6.23 (3.69) | 2.64 (1.76) |
|  | Central nervous system vasculitis | 10 | 9.44 (5.04 - 17.68) | 9.44 (73.61) | 9.23 (5.46) | 3.21 (2.33) |
|  | Miller Fisher syndrome | 8 | 16.5 (8.13 - 33.48) | 16.5 (111.6) | 15.85 (8.77) | 3.99 (3) |
|  | Autoimmune encephalopathy | 8 | 38.42 (18.57 - 79.51) | 38.42 (264.75) | 34.98 (19.03) | 5.13 (4.12) |
|  | Myelin oligodendrocyte glycoprotein antibody-associated disease | 5 | 25.64 (10.36 - 63.41) | 25.63 (110.88) | 24.08 (11.28) | 4.59 (3.37) |
|  | Acute motor axonal neuropathy | 4 | 17.85 (6.55 - 48.67) | 17.85 (60.78) | 17.1 (7.39) | 4.1 (2.77) |
|  | Acute motor-sensory axonal neuropathy | 4 | 8.07 (3 - 21.73) | 8.07 (24.27) | 7.92 (3.46) | 2.99 (1.68) |
|  | Immune-mediated myelitis | 3 | 284.55 (63.68 - 1271.42) | 284.54 (484.37) | 163.03 (46.59) | 7.35 (5.58) |
|  | Meningitis noninfective | 2 | 16.14 (3.92 - 66.47) | 16.14 (27.25) | 15.53 (4.75) | 3.96 (2.24) |
|  | Acute disseminated encephalomyelitis | 2 | 2.36 (0.59 - 9.49) | 2.36 (1.56) | 2.36 (0.74) | 1.24 (-0.44) |
|  | Noninfectious myelitis | 1 | 5.2 (0.72 - 37.39) | 5.2 (3.34) | 5.14 (0.99) | 2.36 (0.29) |
| Nivolumab | Myositis | 430 | 18.44 (16.73 - 20.33) | 18.4 (6679.46) | 17.42 (16.06) | 4.12 (3.98) |
|  | Myasthenia gravis | 353 | 18.47 (16.59 - 20.57) | 18.44 (5496.41) | 17.46 (15.96) | 4.13 (3.97) |
|  | Immune-mediated myositis | 137 | 25.32 (21.27 - 30.14) | 25.3 (2957.22) | 23.47 (20.29) | 4.55 (4.3) |
|  | Meningitis aseptic | 119 | 9.67 (8.06 - 11.6) | 9.66 (896.29) | 9.4 (8.07) | 3.23 (2.97) |
|  | Guillain-Barre syndrome | 106 | 7.98 (6.58 - 9.68) | 7.98 (631.05) | 7.81 (6.64) | 2.96 (2.68) |
|  | Immune-mediated encephalitis | 70 | 92.92 (71.14 - 121.36) | 92.88 (4897.41) | 71.72 (57.36) | 6.16 (5.78) |
|  | Encephalitis autoimmune | 66 | 21.69 (16.9 - 27.84) | 21.68 (1217.25) | 20.33 (16.5) | 4.35 (3.98) |
|  | Immune-mediated myasthenia gravis | 64 | 177.49 (130.55 - 241.3) | 177.42 (7144.69) | 113.27 (87.6) | 6.82 (6.41) |
|  | Dermatomyositis | 63 | 9.02 (7.02 - 11.59) | 9.02 (436.48) | 8.79 (7.13) | 3.14 (2.77) |
|  | Myasthenic syndrome | 55 | 29.86 (22.64 - 39.38) | 29.85 (1399.31) | 27.32 (21.68) | 4.77 (4.37) |
|  | Polymyositis | 45 | 8.89 (6.61 - 11.96) | 8.89 (306.27) | 8.67 (6.76) | 3.12 (2.68) |
|  | Myelitis | 28 | 6.58 (4.52 - 9.56) | 6.58 (129.65) | 6.46 (4.72) | 2.69 (2.15) |
|  | Myasthenia gravis crisis | 22 | 6.12 (4.01 - 9.34) | 6.12 (92.43) | 6.02 (4.23) | 2.59 (1.98) |
|  | Chronic inflammatory demyelinating polyradiculoneuropathy | 21 | 5.89 (3.83 - 9.07) | 5.89 (83.67) | 5.8 (4.04) | 2.54 (1.91) |
|  | Myelitis transverse | 20 | 5.12 (3.29 - 7.97) | 5.12 (65.3) | 5.06 (3.49) | 2.34 (1.7) |
|  | Multiple sclerosis | 19 | 0.13 (0.08 - 0.21) | 0.13 (108.1) | 0.13 (0.09) | -2.91 (-3.56) |
|  | Autoimmune myositis | 16 | 18 (10.87 - 29.8) | 18 (242.8) | 17.07 (11.19) | 4.09 (3.37) |
|  | Necrotising myositis | 13 | 6.28 (3.63 - 10.87) | 6.28 (56.53) | 6.17 (3.9) | 2.63 (1.85) |
|  | Noninfective encephalitis | 13 | 6.75 (3.9 - 11.69) | 6.75 (62.32) | 6.63 (4.18) | 2.73 (1.95) |
|  | Neuromyelitis optica spectrum disorder | 10 | 3.41 (1.83 - 6.36) | 3.41 (16.83) | 3.38 (2.01) | 1.76 (0.88) |
|  | Ocular myasthenia | 9 | 9.51 (4.9 - 18.45) | 9.5 (66.46) | 9.25 (5.31) | 3.21 (2.28) |
|  | Autoimmune encephalopathy | 9 | 35.83 (17.97 - 71.43) | 35.83 (273.16) | 32.22 (18.09) | 5.01 (4.05) |
|  | Miller Fisher syndrome | 9 | 15.27 (7.82 - 29.82) | 15.27 (114.39) | 14.6 (8.34) | 3.87 (2.93) |
|  | Central nervous system vasculitis | 6 | 4.59 (2.05 - 10.27) | 4.59 (16.59) | 4.54 (2.31) | 2.18 (1.08) |
|  | Acute motor axonal neuropathy | 4 | 14.61 (5.36 - 39.83) | 14.61 (48.44) | 14 (6.05) | 3.81 (2.48) |
|  | Acute disseminated encephalomyelitis | 3 | 2.91 (0.93 - 9.07) | 2.91 (3.73) | 2.89 (1.12) | 1.53 (0.08) |
|  | Acute motor-sensory axonal neuropathy | 2 | 3.27 (0.81 - 13.16) | 3.27 (3.12) | 3.24 (1.01) | 1.7 (0.02) |
|  | Immune-mediated myelitis | 1 | 51.75 (6.23 - 429.85) | 51.75 (42.66) | 44.5 (7.57) | 5.48 (3.19) |
|  | Meningitis noninfective | 1 | 6.47 (0.89 - 46.86) | 6.47 (4.53) | 6.36 (1.21) | 2.67 (0.59) |
| Cemiplimab | Myositis | 28 | 36.37 (25.07 - 52.76) | 36.19 (954.76) | 36.06 (26.42) | 5.17 (4.63) |
|  | Myasthenia gravis | 15 | 23.7 (14.27 - 39.36) | 23.64 (324.46) | 23.58 (15.43) | 4.56 (3.84) |
|  | Myasthenic syndrome | 7 | 111.91 (53.1 - 235.84) | 111.77 (759.87) | 110.53 (59.24) | 6.79 (5.76) |
|  | Encephalitis autoimmune | 4 | 39.35 (14.73 - 105.09) | 39.32 (148.8) | 39.17 (17.22) | 5.29 (4) |
|  | Autoimmune myositis | 4 | 137.59 (51.27 - 369.23) | 137.49 (534.58) | 135.62 (59.38) | 7.08 (5.78) |
|  | Immune-mediated myositis | 3 | 16.37 (5.27 - 50.82) | 16.36 (43.2) | 16.34 (6.33) | 4.03 (2.59) |
|  | Meningitis aseptic | 2 | 5.03 (1.26 - 20.11) | 5.02 (6.44) | 5.02 (1.57) | 2.33 (0.66) |
|  | Ocular myasthenia | 2 | 65.8 (16.38 - 264.39) | 65.78 (126.74) | 65.35 (20.41) | 6.03 (4.36) |
|  | Guillain-Barre syndrome | 1 | 2.34 (0.33 - 16.63) | 2.34 (0.77) | 2.34 (0.45) | 1.23 (-0.81) |
|  | Miller Fisher syndrome | 1 | 51.84 (7.26 - 369.98) | 51.83 (49.59) | 51.56 (9.96) | 5.69 (3.64) |
|  | Chronic inflammatory demyelinating polyradiculoneuropathy | 1 | 8.79 (1.24 - 62.44) | 8.78 (6.89) | 8.78 (1.7) | 3.13 (1.09) |
|  | Central nervous system vasculitis | 1 | 24.09 (3.38 - 171.47) | 24.09 (22.07) | 24.03 (4.65) | 4.59 (2.54) |
|  | Polymyositis | 1 | 6.13 (0.86 - 43.53) | 6.13 (4.29) | 6.12 (1.19) | 2.61 (0.57) |
|  | Myasthenia gravis crisis | 1 | 8.71 (1.23 - 61.89) | 8.71 (6.82) | 8.7 (1.69) | 3.12 (1.08) |
| Atezolizumab | Myositis | 173 | 22.82 (19.62 - 26.54) | 22.75 (3517.16) | 22.26 (19.62) | 4.48 (4.25) |
|  | Myasthenia gravis | 95 | 15.16 (12.38 - 18.57) | 15.14 (1235.47) | 14.92 (12.59) | 3.9 (3.6) |
|  | Guillain-Barre syndrome | 63 | 14.96 (11.66 - 19.18) | 14.94 (807.3) | 14.73 (11.96) | 3.88 (3.52) |
|  | Encephalitis autoimmune | 50 | 51.46 (38.72 - 68.39) | 51.41 (2349.49) | 48.92 (38.56) | 5.61 (5.2) |
|  | Dermatomyositis | 32 | 14.38 (10.14 - 20.39) | 14.37 (392.49) | 14.18 (10.59) | 3.83 (3.32) |
|  | Immune-mediated encephalitis | 29 | 104.26 (71.1 - 152.88) | 104.21 (2681.55) | 94.36 (68.5) | 6.56 (6.01) |
|  | Meningitis aseptic | 17 | 4.28 (2.66 - 6.89) | 4.28 (42.53) | 4.26 (2.86) | 2.09 (1.41) |
|  | Myasthenic syndrome | 17 | 27.55 (17.01 - 44.61) | 27.54 (423) | 26.82 (17.92) | 4.75 (4.06) |
|  | Myelitis | 13 | 9.61 (5.57 - 16.6) | 9.61 (99.3) | 9.53 (6.03) | 3.25 (2.48) |
|  | Myelitis transverse | 12 | 9.72 (5.51 - 17.17) | 9.72 (92.96) | 9.63 (5.99) | 3.27 (2.46) |
|  | Immune-mediated myositis | 12 | 6.57 (3.72 - 11.59) | 6.57 (56.25) | 6.53 (4.06) | 2.71 (1.9) |
|  | Polymyositis | 10 | 6.15 (3.3 - 11.45) | 6.15 (42.85) | 6.12 (3.64) | 2.61 (1.74) |
|  | Noninfective encephalitis | 5 | 8.15 (3.38 - 19.66) | 8.15 (31.12) | 8.09 (3.88) | 3.02 (1.83) |
|  | Miller Fisher syndrome | 5 | 26.42 (10.87 - 64.23) | 26.42 (119.11) | 25.76 (12.25) | 4.69 (3.49) |
|  | Autoimmune myositis | 4 | 13.73 (5.12 - 36.82) | 13.72 (46.54) | 13.55 (5.93) | 3.76 (2.46) |
|  | Multiple sclerosis | 4 | 0.09 (0.03 - 0.24) | 0.09 (37.5) | 0.09 (0.04) | -3.49 (-4.78) |
|  | Chronic inflammatory demyelinating polyradiculoneuropathy | 4 | 3.52 (1.32 - 9.39) | 3.52 (7.18) | 3.51 (1.54) | 1.81 (0.52) |
|  | Autoimmune encephalopathy | 3 | 35.29 (11.16 - 111.65) | 35.29 (96.51) | 34.11 (13.01) | 5.09 (3.62) |
|  | Acute motor axonal neuropathy | 3 | 34.47 (10.9 - 109) | 34.47 (94.21) | 33.34 (12.73) | 5.06 (3.58) |
|  | Acute motor-sensory axonal neuropathy | 3 | 15.69 (5.01 - 49.08) | 15.69 (40.6) | 15.46 (5.95) | 3.95 (2.49) |
|  | Ocular myasthenia | 2 | 6.57 (1.63 - 26.38) | 6.57 (9.37) | 6.53 (2.04) | 2.71 (1.03) |
|  | Central nervous system vasculitis | 2 | 4.82 (1.2 - 19.34) | 4.82 (6.03) | 4.8 (1.5) | 2.26 (0.59) |
|  | Myasthenia gravis crisis | 2 | 1.74 (0.43 - 6.97) | 1.74 (0.63) | 1.74 (0.54) | 0.8 (-0.87) |
|  | Meningitis noninfective | 1 | 20.59 (2.84 - 149.16) | 20.59 (18.26) | 20.19 (3.85) | 4.34 (2.25) |
|  | Immune-mediated myasthenia gravis | 1 | 5.65 (0.79 - 40.31) | 5.65 (3.8) | 5.62 (1.09) | 2.49 (0.44) |
|  | Myelin oligodendrocyte glycoprotein antibody-associated disease | 1 | 12.67 (1.76 - 91.08) | 12.67 (10.61) | 12.52 (2.4) | 3.65 (1.58) |
|  | Necrotising myositis | 1 | 1.51 (0.21 - 10.73) | 1.51 (0.17) | 1.51 (0.29) | 0.59 (-1.45) |
|  | Neuromyelitis optica spectrum disorder | 1 | 1.07 (0.15 - 7.63) | 1.07 (0.01) | 1.07 (0.21) | 0.1 (-1.94) |
| Durvalumab | Myositis | 55 | 15.59 (11.95 - 20.33) | 15.56 (743.94) | 15.45 (12.37) | 3.95 (3.56) |
|  | Myasthenia gravis | 54 | 18.71 (14.31 - 24.47) | 18.67 (895.6) | 18.52 (14.8) | 4.21 (3.82) |
|  | Encephalitis autoimmune | 19 | 41.38 (26.28 - 65.16) | 41.35 (734.03) | 40.59 (27.76) | 5.34 (4.69) |
|  | Guillain-Barre syndrome | 17 | 8.72 (5.41 - 14.04) | 8.71 (115.58) | 8.68 (5.83) | 3.12 (2.44) |
|  | Immune-mediated myositis | 15 | 17.97 (10.81 - 29.88) | 17.96 (238.26) | 17.82 (11.65) | 4.16 (3.43) |
|  | Polymyositis | 10 | 13.44 (7.22 - 25.03) | 13.43 (114.37) | 13.36 (7.94) | 3.74 (2.87) |
|  | Myelitis transverse | 9 | 15.89 (8.25 - 30.62) | 15.89 (124.63) | 15.78 (9.11) | 3.98 (3.06) |
|  | Myasthenic syndrome | 8 | 27.91 (13.89 - 56.06) | 27.9 (204.85) | 27.56 (15.37) | 4.78 (3.82) |
|  | Immune-mediated myasthenia gravis | 8 | 102.83 (50.59 - 209.03) | 102.8 (769.83) | 98.17 (54.23) | 6.62 (5.63) |
|  | Dermatomyositis | 6 | 5.82 (2.61 - 12.97) | 5.82 (23.88) | 5.81 (2.97) | 2.54 (1.45) |
|  | Myelitis | 4 | 6.42 (2.4 - 17.12) | 6.42 (18.23) | 6.4 (2.81) | 2.68 (1.39) |
|  | Autoimmune myositis | 3 | 22.41 (7.19 - 69.91) | 22.41 (60.73) | 22.19 (8.57) | 4.47 (3.02) |
|  | Ocular myasthenia | 3 | 21.59 (6.92 - 67.33) | 21.59 (58.32) | 21.38 (8.26) | 4.42 (2.97) |
|  | Immune-mediated encephalitis | 3 | 21.52 (6.9 - 67.1) | 21.52 (58.11) | 21.31 (8.23) | 4.41 (2.96) |
|  | Chronic inflammatory demyelinating polyradiculoneuropathy | 2 | 3.83 (0.96 - 15.35) | 3.83 (4.18) | 3.83 (1.2) | 1.94 (0.27) |
|  | Necrotising myositis | 2 | 6.6 (1.65 - 26.46) | 6.6 (9.48) | 6.58 (2.06) | 2.72 (1.05) |
|  | Miller Fisher syndrome | 2 | 22.73 (5.64 - 91.54) | 22.72 (41.1) | 22.5 (7.01) | 4.49 (2.81) |
|  | Myasthenia gravis crisis | 2 | 3.8 (0.95 - 15.22) | 3.8 (4.12) | 3.8 (1.19) | 1.92 (0.26) |
|  | Neuromyelitis optica spectrum disorder | 2 | 4.7 (1.17 - 18.82) | 4.7 (5.81) | 4.69 (1.47) | 2.23 (0.56) |
|  | Noninfective encephalitis | 1 | 3.54 (0.5 - 25.17) | 3.54 (1.82) | 3.53 (0.68) | 1.82 (-0.22) |
|  | Meningitis aseptic | 1 | 0.55 (0.08 - 3.89) | 0.55 (0.37) | 0.55 (0.11) | -0.87 (-2.91) |
|  | Multiple sclerosis | 1 | 0.05 (0.01 - 0.34) | 0.05 (18.72) | 0.05 (0.01) | -4.37 (-6.41) |
| Avelumab | Myositis | 28 | 44.28 (30.52 - 64.25) | 44.01 (1172.92) | 43.86 (32.12) | 5.45 (4.92) |
|  | Myasthenia gravis | 17 | 32.71 (20.3 - 52.7) | 32.59 (519.22) | 32.51 (21.81) | 5.02 (4.34) |
|  | Immune-mediated myositis | 6 | 39.92 (17.9 - 89.02) | 39.87 (226.6) | 39.74 (20.31) | 5.31 (4.22) |
|  | Guillain-Barre syndrome | 5 | 14.26 (5.93 - 34.3) | 14.25 (61.52) | 14.23 (6.83) | 3.83 (2.65) |
|  | Miller Fisher syndrome | 2 | 126.79 (31.47 - 510.82) | 126.73 (246.88) | 125.42 (39.08) | 6.97 (5.29) |
|  | Ocular myasthenia | 2 | 80.03 (19.92 - 321.58) | 80 (154.99) | 79.48 (24.82) | 6.31 (4.64) |
|  | Myelitis | 2 | 17.87 (4.46 - 71.55) | 17.86 (31.79) | 17.84 (5.59) | 4.16 (2.49) |
|  | Noninfective encephalitis | 2 | 39.56 (9.87 - 158.57) | 39.54 (74.88) | 39.41 (12.33) | 5.3 (3.63) |
|  | Autoimmune myositis | 2 | 83.07 (20.67 - 333.84) | 83.03 (160.98) | 82.47 (25.75) | 6.37 (4.69) |
|  | Dermatomyositis | 2 | 10.8 (2.7 - 43.23) | 10.8 (17.77) | 10.79 (3.38) | 3.43 (1.76) |
|  | Myasthenic syndrome | 2 | 38.54 (9.62 - 154.51) | 38.53 (72.87) | 38.41 (12.02) | 5.26 (3.59) |
|  | Central nervous system vasculitis | 1 | 29.3 (4.12 - 208.55) | 29.29 (27.26) | 29.22 (5.66) | 4.87 (2.82) |
|  | Meningitis aseptic | 1 | 3.05 (0.43 - 21.7) | 3.05 (1.38) | 3.05 (0.59) | 1.61 (-0.43) |
|  | Autoimmune encephalopathy | 1 | 140.03 (19.5 - 1005.66) | 139.99 (136.42) | 138.4 (26.59) | 7.11 (5.05) |
|  | Polymyositis | 1 | 7.45 (1.05 - 52.95) | 7.45 (5.58) | 7.45 (1.44) | 2.9 (0.85) |
|  | Encephalitis autoimmune | 1 | 11.92 (1.68 - 84.74) | 11.92 (9.99) | 11.91 (2.31) | 3.57 (1.53) |
